# Ribonuclease toxin RelE1 inhibits growth of *Mycobacterium tuberculosis* through specific cleavage of the ribosomal anti-Shine–Dalgarno region

**DOI:** 10.1093/nar/gkaf1070

**Published:** 2025-11-17

**Authors:** Xue Han, Izaak N Beck, Moise Mansour, Tom J Arrowsmith, Roland Barriot, Paul Chansigaud, Carine Pagès, Hussein Hamze, Hatice Akarsu, Laurent Falquet, Peter Redder, Xibing Xu, Tim R Blower, Pierre Genevaux

**Affiliations:** Laboratoire de Microbiologie et Génétique Moléculaires (LMGM), Centre de Biologie Intégrative (CBI), Université de Toulouse, CNRS, 31062, Toulouse, France; Department of Biosciences, Durham University, Stockton Road, Durham DH1 3LE, United Kingdom; Laboratoire de Microbiologie et Génétique Moléculaires (LMGM), Centre de Biologie Intégrative (CBI), Université de Toulouse, CNRS, 31062, Toulouse, France; Department of Biosciences, Durham University, Stockton Road, Durham DH1 3LE, United Kingdom; Laboratoire de Microbiologie et Génétique Moléculaires (LMGM), Centre de Biologie Intégrative (CBI), Université de Toulouse, CNRS, 31062, Toulouse, France; Laboratoire de Microbiologie et Génétique Moléculaires (LMGM), Centre de Biologie Intégrative (CBI), Université de Toulouse, CNRS, 31062, Toulouse, France; Laboratoire de Microbiologie et Génétique Moléculaires (LMGM), Centre de Biologie Intégrative (CBI), Université de Toulouse, CNRS, 31062, Toulouse, France; Molecular, Cellular and Developmental Biology Unit (MCD), Centre de Biologie Intégrative (CBI), Université de Toulouse, CNRS, 31062, Toulouse, France; Department of Biology, University of Fribourg & Swiss Institute of Bioinformatics, CH-1700, Fribourg, Switzerland; Department of Biology, University of Fribourg & Swiss Institute of Bioinformatics, CH-1700, Fribourg, Switzerland; Laboratoire de Microbiologie et Génétique Moléculaires (LMGM), Centre de Biologie Intégrative (CBI), Université de Toulouse, CNRS, 31062, Toulouse, France; Laboratoire de Microbiologie et Génétique Moléculaires (LMGM), Centre de Biologie Intégrative (CBI), Université de Toulouse, CNRS, 31062, Toulouse, France; Department of Biosciences, Durham University, Stockton Road, Durham DH1 3LE, United Kingdom; New England Biolabs, 240 County Road, Ipswich, MA 01938, United States; Laboratoire de Microbiologie et Génétique Moléculaires (LMGM), Centre de Biologie Intégrative (CBI), Université de Toulouse, CNRS, 31062, Toulouse, France

## Abstract

Toxin–antitoxin (TA) systems are central to bacterial immunity, genome maintenance, and pathogenicity. Toxins of TA systems use diverse strategies to control bacterial growth and represent attractive therapeutic targets to fight pathogens. In this work, we have investigated the toxic mechanism of the three RelE toxins of *Mycobacterium tuberculosis*, the bacterium responsible for tuberculosis in humans. Structural studies showed that RelBE1, RelBE2, and RelBE3 TA complexes share conserved structural motifs distinct from the RelBE complex of *Escherichia coli*. Although RelE homologs have previously been reported to perform ribosome-dependent messenger RNA (mRNA) cleavage, detection of cleavage products by nEMOTE demonstrated that only RelE3 targets mRNA. In contrast, *in vitro* and *in vivo* analyses using *Mycobacterium smegmatis* and *M. tuberculosis* revealed that RelE1 is a site-specific RNase, able to cleave 16S rRNA from free 30S and formed 70S ribosomes, to release the anti-Shine–Dalgarno region and prevent translation. This stunning mode of action, which is likely shared with RelE2, demonstrates that there is broader diversity for toxic mechanisms within the widespread RelE family.

## Graphical Abstract

**Graphical Abstract:**
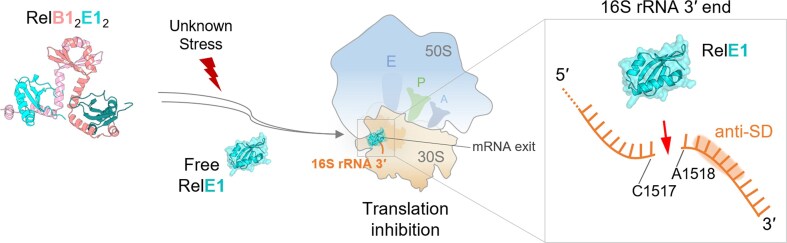

## Introduction

Toxin–antitoxin (TA) systems are small genetic elements involved in the defense against phage infection, in the maintenance of chromosomal regions, plasmids, and other mobile genetic elements, and in bacterial virulence and antibiotic persistence [1–6]. They are ubiquitous in archaeal and bacterial genomes, and are generally stress-responsive [1]. Under lenient growth conditions, the activity or expression of the deleterious toxin is normally inhibited by its counteracting antitoxin and bacterial growth is not detectably affected. However, in the presence of stress, such as phage infection, plasmid loss, or the presence of drugs, the equilibrium between the toxin and the cognate antitoxin can be perturbed in favor of the toxin, leading to toxin activation. Active toxins have been shown to employ diverse strategies to inhibit growth or kill bacteria by targeting essential cellular processes or structures, including translation, DNA replication, metabolism, or the cell envelope [1, 7, 8]. The self-poisoning nature of certain toxins has raised the possibility that such toxins might be used either as activable endogenous antimicrobials, alone or in combination with antibiotics, or as tools to identify new drug targets [9, 10].

Tuberculosis (TB) is a major cause of death and is caused by a single bacterial pathogen, namely *Mycobacterium tuberculosis*. The increasing occurrence of multidrug resistant strains of *M. tuberculosis* represents a major threat to public health and necessitates the development of novel drugs and host-targeted therapies (World Health Organization, www.who.int). Remarkably, the genome of *M. tuberculosis* contains many TA genes (over 80 known TA systems), covering different TA families and uncharacterized pairs [8, 11]. Although largely unexplored, many of these TA systems were shown to be induced under relevant stress conditions [12–14] and several mutations in TA operons exhibited a reduced infection in mice or guinea pigs, suggesting that these systems could contribute to *M. tuberculosis* virulence in humans [15–17]. Notably, many TA system toxins have been shown to be deleterious when overexpressed in *M. tuberculosis, Mycobacterium smegmatis*, or *Escherichia coli*, and studies of their detailed toxic mechanisms are beginning to emerge [8].

*Mycobacterium tuberculosis* encodes six known TA systems with toxins belonging to the RelE-family of ribosome-dependent ribonucleases with conserved RNase T1-like folds. This includes the RelE1, RelE2, RelE3(YoeB), HigB1, HigB2, and HigB3 toxins and their respective RelB1, RelB2, RelB3(YefM), HigA1, HigA2, and HigA3 antitoxins [8]. While RelBE1, RelBE2, YefM/YoeB, and HigBA1 were shown to act as *bona fide* functional TA systems *in vivo* in *M. tuberculosis* (i.e. a deleterious toxin neutralized by co-expression of its cognate antitoxin), both HigB2 and HigB3 toxins did not show any detectable toxicity or RNase activity [18]. Although all six TA operons were shown to be induced or repressed under relevant stress conditions for *M. tuberculosis*, no significant phenotype has been associated with their deletion *in vivo*, except for Δ*higB1*, which shows reduced infection in a guinea pig model (Sharma *et al.*, 2021). A recent analysis of HigB1 toxin targets showed that the toxin binds at the ribosomal A site to cleave messenger RNA (mRNA) during translation (similar to the canonical *Escherichia coli* RelE) and identified over twenty transcripts that are targeted by the toxin *in vivo* [18]. The RelE3(YoeB) toxin, which is mildly toxic when compared with HigB1, RelE1, or RelE2 was also shown to act as an RNase [19, 20]. Both RelE1 and RelE2 are acutely toxic, very similar in sequence (∼54% identity) [21] and were proposed to have redundant functions in *M. tuberculosis* [20–22]. To date, the cellular targets and the toxic mechanism of these two toxins remain unknown.

In this work, we investigate the toxic mechanism of the RelE1 toxin of *M. tuberculosis*. We show that RelE1 is acutely toxic in mycobacteria but not in *E. coli*, suggesting host specificity. Furthermore, we solved the crystal structure of the RelE1–RelB1 complex at 2.20 Å resolution and showed that it forms a heterotetrameric complex in which the RelB1 antitoxin likely displaces the C-terminal α-helix of the partner RelE1 toxin, and identified key residues of the catalytic region that are essential for its activity. We show that although RelE1 inhibits translation *in vitro*, it does not act as a ribosome-dependent RNase toxin that cleaves mRNA during translation, as observed for HigB1 or other RelE toxins [18, 23]. In contrast, we demonstrate here that RelE1 affects translation by specifically cleaving the extreme 3′ end of the 16S ribosomal RNA (rRNA) within the 30S ribosomal subunit, immediately upstream of the anti-Shine–Dalgarno (SD) sequence. The impacts of this novel toxic mechanism on *M. tuberculosis* growth control and its conservation among RelE-like toxins are discussed.

## Materials and methods

### Bacterial strains and culture conditions

*Escherichia coli* strains DH5α (Invitrogen), BL21(λDE3) AI (Novagen), Rosetta 2 pLysS (Novagen), and DLT1900 [24], *M. smegmatis* strains mc^2^155 (strain ATCC 700084) and mc^2^155 *rnj102* [25], and *M. tuberculosis* mc^2^ 6230 (*Mtb* H37Rv ∆*RD1* ∆*panCD*) [26] have been published. The mc^2^ 6230 △*relBE1:: ZeoR* mutant strain was constructed by allelic exchange using recombineering, as previously described [27]. In this case, ∼0.5-kb DNA fragments flanking the *relBE1* operon were amplified by polymerase chain reaction (PCR) using primer pairs relBE1_upstream_For/relBE1_upstream_Rev and relBE1_downstream_For/relBE1_downstream, and *M. tuberculosis* H37Rv genomic DNA as template. Zeocin-Resistance cassette with oligonucleotides introducing *dif* site variants (Zeo^R^) on each side was PCR amplified using primers relBE1_ZeoR_For and relBE1_ZeoR_Rev. The Zeo^R^ cassette was inserted within the two *relBE1* flanking regions of homology by a two-step fusion PCR. The resulting linear DNA was electroporated into *M. tuberculosis* mc^2^6230 strain containing plasmid pJV53K (KanR), as described [28]. *Mycobacterium tuberculosis* zeocin-resistant clones were selected, the position of the insert into the genome was PCR verified and DNA sequenced.

*Escherichia coli* strains were grown at 37°C in Luria-Bertani broth (LB) and supplemented with kanamycin (Km, 50 μg ml^−1^), ampicillin (Ap, 50 μg ml^−1^), streptomycin (Sm, 100 μg ml^−1^), isopropyl-β-D-thiogalactopyranoside (IPTG, 1 mM), L-arabinose (L-ara, 0.1% w/v), or D-glucose (glu, 0.2% w/v) when necessary.*Mycobacterium smegmatis* mc^2^155 strains were grown at 37°C in LB supplemented with 0.05% Tween 80 (Sigma–Aldrich) and when necessary, with Km (10 μg ml^−1^) or Sm (25 μg ml^−1^). *Mycobacterium tuberculosis* strains were grown at 37°C in 7H9 medium (Middlebrook 7H9 medium, Difco) supplemented with 10% oleic acid-albumin-dextrose-catalase (OADC, Difco) and 0.05% Tween 80, or on complete 7H11 solid medium (Middlebrook 7H11 agar, Difco) supplemented with 10% OADC. When necessary, media were supplemented with Sm (25 μg ml^−1^), zeocin (Zeo, 25 μg ml^−1^), or anhydrotetracycline (Atc, 100 or 200 ng ml^−1^) [29].

### Plasmid constructs

Plasmids pMPMK6 [30], pGMC [31], pGMC-HigB1 [18], pETDuet-1 and pET15b (Novagen), and pET20b-HigB1 [32] have been described. The primers used to construct the plasmids are described in Supplementary Table S1.

Plasmids pMPMK6–RelE1, RelE2, and RelE3(YoeB) were constructed as follows. The *rv1246c, rv2866*, and *rv3358* genes were PCR amplified from the *M. tuberculosis* H37Rv genome using primers Rv1246c EcoRI-For and Rv1246c HindIII-Rev, Rv2866 EcoRI-For and Rv2866 HindIII-Rev, and Rv3358 EcoRI-For and Rv3358 HindIII-Rev. The obtained fragments were cloned as EcoRI/HindIII fragments into EcoRI/HindIII-digested pMPMK6, respectively.

To obtain plasmid pGMC–RelE1, RelBE1, RelE2, and RelE3/YoeB, after PCR amplification of *rv1246c (relE1), rv1247c-1246c (relBE1), rv2866 (relE2)*, and *rv3358(RelE3/YoeB)* using *the M. tuberculosis* H37Rv genome as template with primers pGMC–RelE1 For and pGMC–RelE1 Rev, pGMC–RelB1 For and pGMC–RelE1 Rev, pGMC–RelE2 For and pGMC–RelE2 Rev, pGMC–RelE3(YoeB) For and pGMC–RelE3(YoeB) Rev, *relE1, relE2*, and *relE3(YoeB)* genes were cloned into linearized pGMC plasmid respectively by homologous recombination using the In-Fusion HD Cloning Kit (Takara Bio). To construct plasmid pGMC–RelE1^B1^, a PCR fragment containing *rv1247c* (*relB1*) gene with its upstream promoter region (250 bp) was amplified from *M. tuberculosis* H37Rv genomic DNA using primers pGMC–RelE1–RelB1 For and pGMC–RelE1–RelB1 Rev, and introduced by In-Fusion into linearized pGMC–RelE1 plasmid. Plasmids pGMC–RelE1^B1^ with R17A, R21A, K47A, R50A, S58A, R60A, R61A, R65A, R85A, or Y89A substitutions were constructed by QuikChange site-directed mutagenesis using appropriate primers and pGMC–RelE1^B1^ as template.

To generate plasmid pETDuet–RelB1_His_, *rv1247c* was PCR amplified from the *M. tuberculosis* H37Rv genome using primers pETduet–RelB1His For and pETduet–RelB1His Rev, and cloned as a BamHI/HindIII fragment into BamHI/HindIII-digested pETDuet-1. For pETDuet–RelB1_His_–RelE1, *relE1* was PCR-amplified from pGMC–RelE1 and cloned as an NdeI/AvaI fragment into NdeI/AvaI-digested pETDuet–RelB1_His_. Plasmid pETDuet–RelB1_His_–RelE1^R65A^ was constructed by QuikChange site-directed mutagenesis using appropriate primers and pETDuet–RelB1_His_–RelE1 as template. Plasmid pETDuet-YefM_His_-YoeB was obtained as follows. The *rv3357 (relB3, yefM)* gene encoding the RelB3(YefM) antitoxin with a C-terminal 6xHis-tag was PCR amplified from *M. tuberculosis* H37Rv genomic DNA using primers pETduet-YefM_His_ For and pETduet-YefM_His_ Rev, and cloned as an EcoRI/HindIII fragment into EcoRI/HindIII digested pETDuet-1. The *rv3358 (relE3, yoeB)* gene then was PCR-amplified and cloned as an NdeI/BglII fragment into NdeI/BglII-digested pETDuet-YefM_His_. Plasmid pTRB638 used to over-express RelBE1 for crystallization is a pET-DUET1 derivative, expressing RelB1 and His-SUMO–RelE1, constructed by Genscript. To construct pET15b–RelE1, *rv1246c* was PCR amplified from the *M. tuberculosis* H37Rv genome using primers RelE1 NdeI For and RelE1 BamHI Rev and cloned into pET15b after digestion with NdeI and BamHI enzymes. Plasmids pET15b–RelE1 with R65A substitution was constructed by QuikChange site-directed mutagenesis using appropriate primers and pGMC–RelE1B1 as template. All the plasmids constructed in this work have been verified by sequencing.

### Western blot analysis

Strain BL21(λDE3) AI transformed with pET15b–RelE1 or pET15b–RelE1^R65A^ was grown to an OD_600_ of ∼0.4 at 37°C. L-ara (0.2%) was added and cultures were incubated overnight at 22°C. Cells were then centrifuged at 5000 × *g* for 10 min at 4°C, pellets were washed with 3 ml lysis buffer (25 mM equimolar solution of Na_2_HPO_4_/NaH_2_PO_4_; 200 mM NaCl; 20 mM imidazole, pH 8.0) and resuspended in 7 ml lysis buffer supplemented with one ethylenediaminetetraacetic acid (EDTA)-free Protease Inhibitor tablets (Roche, to 20 ml) and benzonase 25 U ml-1 (Sigma–Aldrich). Lysis was performed using the One-shot cell disrupter at 1.5 Kbar (One shot model, Constant Systems Ltd) and lysates were centrifuged for 30 min at 30 000 × *g* at 4°C. Proteins [1 μl of lysate supernatant; 9 μl of lysis buffer; 3.3 μl of 4 × sodium dodecyl sulphate (SDS) loading buffer] were then separated on Mini-Protein TGX gels (Bio-Rad) by sodium dodecyl sulphate–polyacrylamide gel electrophoresis (SDS–PAGE) and transferred to polyvinylidene difluoride membrane (Bio-Rad) using the Trans-Blot^®^ TurboTM transfer system (Bio-Rad). Membrane was blocked for 1 h at room temperature (RT) in 5% (w/v) nonfat dry milk in PBS containing 0.05% (v/v) Tween 20. Primary antibody used in this study was anti-His antibody (QIAGEN, dilution 1:1000). Horseradish peroxidase-conjugated mouse IgG (Promega, 1:2500) was used as a secondary antibody. Blots were developed by chemiluminescence using Clarity Western ECL substrate (Bio-Rad) with the ChemidocTM Touch imaging system (Bio-Rad) and analyzed with the Image Lab software (Bio-Rad).

### Protein expression and purification

Purified proteins were obtained as follows. To purify RelE1 and RelE1^R65A^, strain BL21(λDE3) AI transformed with pETDuet–RelB1_His_–RelE1 or pETDuet–RelB1_His_–RelE1^R65A^ was grown to an OD_600_ of ∼0.4 at 37°C, 0.2% L-ara was added and the culture incubated overnight at 22°C. Cultures were centrifuged at 5000 × g for 10 min at 4°C, pellets were washed with 3 ml lysis buffer and resuspended in 7 ml lysis buffer (25 mM equimolar solution of Na_2_HPO_4_/NaH_2_PO_4_; 200 mM NaCl; 20 mM imidazole pH 8.0) supplemented with one EDTA-free Protease Inhibitor tablets (Roche, to 20 ml) and benzonase 25 U ml^−1^ (Sigma–Aldrich). Lysis was performed using the One-shot cell disrupter at 1.5 Kbar (One shot model, Constant Systems Ltd) and incubated for 2 hours on ice. Lysates were centrifuged for 30 min at 30 000 × *g* at 4°C and the resulting supernatants were gently mixed with Ni-NTA Agarose beads (Qiagen) pre-equilibrated with lysis buffer, at 4°C for 30 min in a 10 ml poly-prep column (Bio-Rad). The column was then stabilized for 10 min at 4°C, washed six times with 10 ml of lysis buffer and two times with 10 ml of wash buffer (25 mM equimolar solution of Na_2_HPO_4_/NaH_2_PO_4_; 200 mM NaCl; pH 8.0), and RelE1 and RelE1^R65A^ proteins were eluted under denaturing condition with 400 µl elution buffer (25 mM equimolar solution of Na_2_HPO_4_/NaH_2_PO_4_; 200 mM NaCl; 6M guanidinium chloride). Elutions were collected and D-Tube^TM^ Dialyzer Midi (Molecular weight cutoff [MWCO] 3.5 kDa, Millipore) were used to exchange buffer (25 mM HEPES, ph 7.5, 200 mM NaCl, 2 mM MgCl_2_, 1 mM dithiothreitol [DTT]). Proteins were concentrated using Vivaspin^®^ 6 columns with 5000 MWCO PES membrane (Sartorius) and used directly in subsequent experiments. The YoeB protein purification procedure was identical to that of RelE1, except that the plasmid used was pET-DUET-YefM_His_-YoeB. HigB1 protein purification was carried out as described [18].

For crystallization, RelB1 and His-SUMO–RelE1 were co-expressed from Rosetta 2 pLysS cells transformed with pTRB638. Cells were grown at 37°C with shaking at 180 rpm to an OD_600_ of ∼0.6, at which point the incubation temperature was reduced to 18°C and IPTG was added to 1 mM final concentration. Cells were grown for a further 16 h at 18°C with shaking at 160 rpm. Bacterial cells were pelleted from liquid culture by centrifugation at 4200 × *g* for 30 min at 4°C. Cell pellets were resuspended in lysis buffer A500 [20 mM Tris base, pH 8.0, 500 mM NaCl, 30 mM imidazole pH 8.0, 10% (vol vol^−1^) glycerol], and sonicated using a Vibracell™ VCX500 ultrasonicator with medium tip (Sonics) for a total of 2 min (10 s on/10 s off). The sonicated sample was centrifuged at 20 000 × *g* for 1 h at 4°C to isolate the soluble fraction from cell debris. The soluble fraction was passed through Ni-NTA His-Trap^TM^ HP 5 ml columns (Cytiva) at 2 ml min^−1^. A 10-column volume (cv) wash step was performed using lysis buffer A500. The column was then washed with a further 5 cv A100 [20 mM Tris base, pH 8.0, 100 mM NaCl, 10% (vol vol^−1^) glycerol]. The sample was eluted directly on to a pre-equilibrated HiTrap Q HP anion exchange column (Cytiva) with 10 cv B100 [20 mM Tris base, pH 8.0, 100 mM NaCl, 250 mM imidazole, pH 8.0, 10% (vol vol^−1^) glycerol] before washing again in A100 to remove the high imidazole. The anion exchange column was then subjected to an increasing salt gradient using the Åkta™ system, titrating in high salt buffer C1000 [20 mM Tris base, pH 8.0, 1000 mM NaCl, 10% (vol vol^−1^) glycerol] until a final salt concentration of 600 mM NaCl was achieved. 2 ml fractions were collected and analysed by SDS–PAGE. Fractions containing the protein of interest were pooled and 0.5 mg of SENP (sentrin protease) was added to cleave the His-SUMO tag. The sample was rolled overnight at 4°C. In the morning, precipitated protein was pelleted by centrifugation at 20 000 × *g* for 20 min at 4°C and the supernatant was collected and concentrated in a 10 kDa cut-off centrifugal concentrator (Sartorius) to 2 ml. The 2 ml sample was injected into a 2 ml capillary loop on the Åkta™ Pure system before fractionation by size exclusion chromatography using a Sephacryl S-200 column (Cytiva). The column was pre-equilibrated in sizing column buffer, S500 [50 mM Tris base, pH 8.0, 500 mM KCl, 10% (vol vol^−1^) glycerol]. S500 was used for storage of the final protein product at −80°C.

### Protein crystallization and structure determination

RelBE1 protein for crystallography was dialyzed into buffer X (20 mM Tris base, pH 8.0, 150 mM NaCl, 2.5 mM DTT) and concentrated to 12 mg ml^−1^ for initial trials. Commercially available 96-well crystallization screens (Molecular Dimensions) were used to assay preferred crystallization conditions. Sitting drop crystallization trials were set-up using an SPT Labtech Mosquito^®^ crystal robot. Each condition was tested with a 2:1 and 1:1 ratio of protein to mother-liquor on the crystallization stage. Crystal screens were left at 18°C. Early crystal screens resulted in many conditions with high levels of precipitation, or what appeared to be micro-crystals, even when incubated at 4°C. To slow the rate of crystallization the crystal buffer was supplemented with 5% (vol vol^−1^) glycerol to give buffer X-5 [20 mM Tris base, pH 8.0, 150 mM NaCl, 2.5 mM DTT, 5% (vol vol^−1^) glycerol], and the starting RelBE1 concentration was reduced to 8 mg ml^−1^; 4 M ammonium acetate, 0.1 M Bis–Tris propane (pH 7.0) was subsequently identified as the best ‘hit’, yielding small diamond-like crystals. For optimization, the drop size was increased to 2 μl in a 1:1 ratio of protein to mother liquor. Ammonium acetate concentrations above 2.5 M produced incredibly durable hexagonal bifrustum shaped RelBE1 crystals, while reducing the ammonium acetate below 2 M resulted in needle shaped RelBE1 crystals. For harvesting, mother liquor from the appropriate condition and 100% glycerol were mixed in a ratio of 1:1 and an equal volume of this mixture was added to the sitting drop, prior to harvesting, snap-freezing in liquid nitrogen, and storage in a sample puck.

Diffraction data were collected at Diamond Light Source on microfocus beamline i04 (Table 1). Twelve 360° datasets were collected from a single RelBE1 crystal and merged using iSpyB (Diamond Light Source). Initial data processing was automated by Diamond Light Source iSpyB using the X-ray image integration programs Xia2 and Xia2-DIALS [33]. Image integration and space group selection were carried out manually using the same programs as well as Mosflm [34]. Diffraction data were processed with XDS [35], and then AIMLESS from CCP4 [36] was used to corroborate the spacegroup. The crystal structure of RelBE1 was solved by molecular replacement using PHASER and the starting model 3G5O split into individual protomers RelB1 and RelE1 then input as individual assemblies. The solved starting model for RelBE1 was built in REFMAC [37] and BUCCANEER [38]. The model was then iteratively refined and built using PHENIX and COOT [39], respectively. The quality of the final model was assessed using COOT and the wwPDB validation server [40]. Structural figures were generated using PyMol (Schrödinger).

**Table 1.** Values in parentheses are for highest-resolution shell

	RelBE1
PDB ID code	9G12
Number of crystals	12
Beamline	Diamond I04
**Data collection**	
Wavelength, Å	0.9793
Space group	I 21 21 21
Cell dimensions	
*a, b, c* (Å)	88.99 98.54 97.34
α, β, γ (°)	90 90 90
Resolution (Å)	65.68–2.20 (2.279–2.20)
*R*_merge_	0.016 (0.849)
*R*_meas_	0.023 (1.201)
CC_1/2_	1.0 (0.536)
*I* / s*I*	20.9 (0.4)
Completeness (%)	99.00 (91.48)
Redundancy	1.0 (1.0)
**Refinement**	
Resolution (Å)	2.20
No. of reflections	22105
Unique reflections	22101 (2193)
*R*_work_	0.252
*R*_free_	0.279
No. of atoms	2686
Protein	2681
Ligand/ion	1
Water	4
Average B-factors	78.63
Protein	78.62
Ligand/ion	100.88
Water	79.70
Root mean square (r.m.s.) deviations	
Bond lengths (Å)	0.007
Bond angles (°)	0.97
Ramachandran favored (%)	96.12
Ramachandran allowed (%)	3.88
Ramachandran outliers (%)	0.0
Rotamer outliers (%)	0.72
Clashscore	8.79

### Ribosome purification

Ribosomes were purified from 1 l of *M. smegmatis* mc^2^155 culture grown at 37°C in LB. When the culture reached an OD_600_ of 1.0, cells were pelleted by centrifugation at 5000 × *g* for 10 min at 4°C, washed with 14 ml lysis buffer (20 mM Tris–HCl, pH 7.5, 10.5 mM MgOAc, 100 mM NH_4_Cl, 0.5 mM EDTA, and 3 mM 2-mercaptoethanol), resuspended in 16 ml lysis buffer and lysed using the One-shot cell disrupter at 1.3 Kbar three times. The lysate was then clarified by centrifugation at 20 000 × *g* for 10 min and at 25 000 × *g* for 1 h at 4°C. The supernatant was next layered 1:1 (v:v) over a high-salt sucrose cushion buffer (20 mM Tris–HCl, pH 7.5, 10.5 mM MgOAc, 500 mM NH_4_Cl, 0.5 mM EDTA, 3 mM 2-mercaptoethanol, 1.1 M sucrose) in 6 Quick-Seal Centrifuge Tubes (Beckman Coulter, 5.1 ml). After ultracentrifugation at 100 000 × *g* for 22 h at 4°C, the resulting ribosome pellets were washed twice with 100 µl storage buffer (10 mM Tris–HCl, pH 7.5, 10.5 mM MgOAc, 60 mM NH_4_Cl, 3 mM 2-mercaptoethanol) and dissolved in 8 µl of storage buffer overnight at 4°C. Ribosomes were flash frozen in liquid nitrogen and stored at −80°C until further use. To dissociate the 70S ribosome to respective subunits, 30S and 50S, the concentration of MgOAC was reduced from 10.5 to 5 mM and KCl was added to a concentration of 500 mM followed by incubation on ice for 1 h 30 min. Ribosome and subunits mixture was layered on a 10%–30% sucrose gradient prepared in Tp gradient buffer (50 mM Tris–HCl, pH 7.5, 5 mM MgCl_2_, 500 mM KCl), and centrifugation was carried out at 186 000 × *g* for 3.2 h. The peaks corresponding to 50S and 30S were collected and concentrated with Vivacon^®^ 2 centrifugal devices separately. The concentration was estimated by measuring the absorbance at 260 nm. Aliquots of subunits were flash frozen in liquid nitrogen and stored at −80°C until further use.

### Cell-free transcription/translation system *in vitro*

To investigate whether RelE1 inhibits translation *in vitro, E. coli* and *M. smegmatis* cell-free transcription/translation systems based on PURE system (NEB, *E. coli* PURE) were used. *Escherichia coli* based cell-free transcription-translation *in vitro* assays were performed as described [18, 41]. Briefly, DNA of *cspA* (*rv3648c)* 204 bp was amplified by PCR using primers containing T7 promoter and terminator and added at a final concentration of 20 ng µl^−1^ to the *E. coli* PURE system. Protein synthesis was performed at 37°C for 2 h in the presence of 0.6 μCi μl^−1^ of [^35^S]-Methionine with or without toxins (10 µM). The reaction was stopped by placing on ice and samples were then separated by SDS–PAGE on 4%–20% Mini-Protean TGX gels (Bio-Rad) for 30 min at 200 V. Gels were fixed in 10% acetic acid/40% methanol (v/v) for 30 min and proteins were visualized using a Typhoon phosphorimager (GE Healthcare) and Multigauge software (Fuji).

*Mycobacterium smegmatis*-based hybrid *in vitro* transcription/translation system was performed as follows. Active ribosome fractions of *M. smegmatis* were purified and added to PURExpress^®^ Δ Ribosome Kit (NEB) at a final concentration of 2.5 µM. Freshly purified toxins were added to the system at a final concentration of 10 µM. T7-DNA templates of *cspA* (*rv3648c*) 204 bp, *gfp* 717 bp and *gly* (*MSMEG_6630*) 351 bp were added at a final concentration of 20 ng μl^−1^ to the *M. smegmatis* transcription/translation system. Similar experiments were also performed by directly adding purified mRNA. In this case, mRNA of *cspA* and *gfp* were *in vitro* transcribed with T7 RNA polymerase (NEB) and purified and then added to cell free *M. smegmatis* translation reactions at a final concentration of 60 ng μl^−1^. Protein synthesis was performed at 37°C for 2 h in the presence of 0.6 μCi μl^−1^ of [^35^S]-Methionine and the reaction was stopped by placing on ice. Protein synthesis was visualized as described above for the *E. coli* PURE assay.

In addition to the monitoring of protein synthesis, total RNA from *in vitro* translation reactions were extracted for subsequent experiments including mRNA reverse transcription, northern blotting assay and RNA sequencing. In this case, cold DEPC-H_2_O (pH 7.0) was added to each *in vitro* translation reaction to a total volume of 100 µl, followed by 300 µl of TRIzol (Sigma–Aldrich) and 100 µl of cold chloroform. After mixing for 30 s and centrifugation at 20 000 × *g* at 4°C for 15 min, the aqueous phase was transferred to a new centrifuge tube and 100 µl of isopropanol was added to precipitate the RNA. RNA was washed twice with 75% ethanol, then dissolved in DEPC-H_2_O and stored at −80°C until further use.

### Primer extension

For primer extension 2 µg of purified RNA from the *M. smegmatis* transcription/translation extracts, 0.05 µM [^32^P]-labeled *cspA, gfp*, or *gly* extension primer and 1 mM dNTPs were mixed in a 10 µl volume, incubated at 65°C for 5 min, and chilled on ice for 2 min. Finally, 10 µl of 2× buffer [mix 4 µl 5× ProtoScript II RT (NEB), 2 µl 0.1 M DTT, 8 units RNasin^®^ Plus Ribonuclease Inhibitor (Promega), 200 units of ProtoScript II RT Enzyme (NEB) in 10 µl] were added and incubated at 48°C for 1 h. The resulting complementary DNA (cDNA) were mixed with RNA loading dye, loaded on a 6% polyacrylamide gel containing 7 M urea, separated at 200 V for 2 h 30 min, and imaged by autoradiography using Typhoon phosphorimager (GE Healthcare) and Multigauge (Fuji Film). Note that ^32^P labeling of the primer was performed using T4 Polynucleotide Kinase (10 U, NEB) at 37°C for 1 h in the presence of extension primer (0.5 µM final concentration) and 2.5 µCi µl^−1^ of ATP, [γ-^32^P]. The labeled primer was purified with Bio-Spin^®^ 6 Columns (Bio-Rad).

### RelE1 overexpression in *vivo* and preparation of RNA samples

To investigate whether RelE1 has an effect on RNA processing *in vivo*, RelE1 was overexpressed in both *M. smegmatis* and *M. tuberculosis*, and total RNA were extracted for subsequent experiments including northern blotting assay and RNA sequencing. In this case, *M. smegmatis* was transformed with pGMC, pGMC–RelE1–RelB1, or pGMC–RelE1^R65A^–RelB1 and grown for 3 days at 37°C. Cell cultures were transferred to fresh LB medium and grown at 37°C until OD_600_ 0.1. Atc (100 ng ml^−1^) inducer was added to the cultures and equivalent amounts of cells were pelleted by centrifugation at 3500 × *g* for 10 min at 4°C at different time points after incubation at 37°C for preparation of total RNA. For each sample, pellet was re-suspended in 1 ml cold TRIzol and transferred into a 2 ml Safe-Lock tube with 35–50 mg acid-washed glass beads [425–600 μm (30–40 U.S. sieve), Sigma–Aldrich]. The cells were disrupted using a bead-beater disrupter (Precellys^®^ 24 Touch, Bertin Technologies) 3 × 1 min ON–30 s OFF–1 min ON. The lysate was centrifuged at 20 000 × *g* at 4°C for 2 min. The supernatant was transferred to a new RNase-free Eppendorf tube and 400 µl of cold chloroform was added; the tube was gently shaken for 30 s immediately and placed on ice for 15 min. The sample was centrifuged at 20 000 × *g* for 20 min at 4°C and the aqueous phase was transferred into a new RNase-free Eppendorf tube and 500 µl of cold isopropanol was added. The sample was mixed immediately and put at −20°C overnight to precipitate RNA. RNA was washed twice with 75% ethanol, then dissolved in DEPC-H_2_O and stored at −80°C until further use. In the case of *M. tuberculosis* mc^2^ 6230, the strain was transformed with pGMC or pGMC–RelE1 and grown at 37°C until OD_600_ reached 0.4. Atc (200 ng ml^−1^) was added and equivalent amounts of cells were pelleted by centrifugation at 3500 × *g* for 10 min at 4°C after 0, 4, or 24 h. Cell pellets were resuspended in 1 ml of TRIzol and cells were disrupted using bead-beater disrupter. Lysates were centrifuged for 2 min at 20 000 × *g* at 4°C and TRIzol extracts were collected and conserved for at least 48 h at −80°C before being transferred out of the BSL2 laboratory for further total RNA isolation as described for *M. smegmatis* RNA preparation.

### Northern blotting assay

Northern blotting experiments were performed as described [42]. Briefly, equal amounts of total RNA (usually 4 μg for analysis of rRNA *in vivo* or 2 μg for the rRNA *in vitro M. smegmatis* transcription/translation system) were mixed with five volumes of Glyoxal loading buffer (prepared by mixing the following: 6 ml dimethyl sulfoxide [DMSO], 2 ml deionized glyoxal, 1.2 ml 10× BPTE buffer (100 mM PIPES, 300 mM Bis–Tris, 10 mM EDTA), 600 μl 80% glycerol, 40 μl 10 mg ml^−1^ ethidium bromide]. The samples were heated for 1 h at 55°C and RNA were separated by electrophoresis on 1.2% agarose gels in 1× BPTE. After electrophoresis, the gels were (i) rinsed for 10 min with ultrapure MilliQ H_2_O, (ii) soaked for 20 min at RT in 75 mM NaOH with gentle shaking to partially hydrolyze RNA, (iii) rinsed 2 × 5 min with ultrapure MilliQ H_2_O, (iv) soaked 2 × 15 min at RT in (0.5 M Tris–HCl, pH 7.4, 1.5 M NaCl) with gentle shaking to neutralize the pH, (v) rinsed for 10 min with ultrapure MilliQ H_2_O, (vi) soaked 2 × 10 min at RT in 10× saline-sodium citrate (SSC) buffer with gentle shaking. RNAs were then transferred over night to Amersham Hybond-N + membranes (GE Healthcare) by capillarity with 10× SSC transfer buffer. Membranes were then exposed to 0.125 joules of 365 nm ultraviolet (UV) rays to crosslink RNA on the membranes. Membranes were then hybridized with [^32^P]-labeled oligonucleotide probes using the ROTI^®^Hybri-Quick buffer (ROTH). Radioactive membranes were exposed to PhosphorImager screens and signals were revealed using Typhoon imager (GE Healthcare). The sequences of the probes used to detect rRNA are described in Supplementary Table S1.

### nEMOTE analysis

The nEMOTE analysis was performed to investigate whether mRNA was targeted by RelE1, RelE2, or RelE3(YoeB) *in vivo. Mycobacterium smegmatis* WT or *∆rnJ* transformed with pGMC vector, pGMC–RelE1^B1^, pGMC–RelE2, or pGMC–RelE3(YoeB) were grown at 37°C until OD_600_ reached 0.4. Atc (100 ng ml^−1^) was added to induce toxins for 3 h, equivalent amounts of cells were pelleted and total RNA isolation was performed as described [18]. The nEMOTE library was performed as described [18, 43]. Briefly, 8 µg of RNA were first incubated with XRN-1 (NEB) for 4 h to digest mono-phosphorylated RNA. RNA was then split into two pools, one pool of RNA was both phosphorylated with polynucleotide kinase (NEB) and ligated to Rp8 oligo, the control pool of RNA was only ligated to Rp8. The Rp8 oligo that we ligate to the 5′-OH ends of the RNA contains a unique molecular identifier (a series of random nucleotides), which allows us to tag each ligation event with its own unique barcode. Reverse transcription was performed using DRNA primer and ProtoScript II RT enzyme. Finally, PCR amplification was performed with barcode primers and Illumina adaptator A and B primer using Q5 Polymerase Hot-Start (NEB).

Raw sequencing reads were first filtered and trimmed using the emoteStepI method from a Perl program called EMOTE-conv [44]. The trimmed reads are mapped against the *M. smegmatis* genome (NC_008596.1) with bowtie (version 1.2) [45] using the parameters -a -best -strata -v1. The obtained alignment file is submitted to the emoteStepII step from EMOTE-conv in order to compute an annotated coverage table corresponding to the number of reads per position per EMOTE barcode. The unique molecular identifier that was included in the Rp8 oligo allow us to detect when the cDNA from a single ligated RNA molecule is represented by one or more different illumina reads (this is possible due to the PCR amplifications in the library preparations). Thus, all identical reads with the same unique molecular identifier are only counted as a single RNA cleavage event. Downstream analysis and plots are performed in R (version 3.4.4, running under Ubuntu 14.04.6). We set a very conservative cut-off for what was considered a true toxin-dependent cleavage event: The genomic positions where the 5’ OH-ends of at least 10 independent RNA molecules were detected for at least one test sample (toxin + PNK) and where no 5’OH-ends were detected in all three negative control samples (toxin-PNK, vector + PNK, and vector-PNK). The putative cut-site motif is plotted with the R package ggseqlogo (version 0.1).

### Incubation assay of RelE1 with 70S, 30S and purified 16S rRNA in *vitro*

In order to observe 16S rRNA cleavage by RelE1, incubation of the toxin with purified 70S, 30S ribosomal subunits, or free 16S rRNA was performed as follows; 2.5 µM of 70S ribosome, 2 ng of 30S subunits, and 2 ng of RNA extracted from *M. smegmatis* transcription/translation system were incubated with or without 10 µM of toxin, respectively, for 2 h at 37°C. RNA was then extracted using TRIzol and isopropanol precipitation. RNA was dissolved in DEPC-H_2_O and kept at −80°C until further use.

### RNase H digestion assay

An RNase H-based method was applied to identify the 3' end of 16S rRNA cleaved by RelE1. RNA extracts from (i) *in vitro* transcription-translation assay, (ii) *in vivo M. tuberculosis ∆relBE1* with or without RelE1 overexpression for 4 h or 24 h, (iii) *in vivo M. smegmatis* with or without RelE1 overexpression for 1 h or 3 h, and (iv) *in vitro* 70S ribosomes/30S subunits/16s rRNA incubation assay were first incubated with an RNA/DNA/RNA reverse probe hybridizing in the 180 nt from 3′ end of 16S rRNA and then RNase H (NEB) was used to digest the RNA samples to obtain an RNA fragment about 180 nt from the 3′-end of 16S rRNA, which could subsequently be sequenced. In this case, for each RNA sample, 1 µg of RNA (8.5 µl) were denatured at 95°C for 5 min with the RNA/DNA/RNA reverse probe (16S_RNaseH_ probe_1: 5′-ACGUAUUCACCGCAGCGTTGCUGAUCUGCGAUUAC-3′; 0.5 µl at 100 mM). After annealing by cooling down to room temperature for 10 min, the reaction mixture was diluted to 30 µl with a reaction mix containing 1 × RNase H reaction buffer, 66 µM DTT, 20 U RNasin^®^ Plus Ribonuclease Inhibitor and 50 U RNase H (New England Biolabs) or an equal volume of water for the control group, and incubated at 37°C for 30 min. The reaction was then blocked by addition of 0.3 M sodium acetate, pH 5.2 and 0.2 mM EDTA, and the RNA were recovered by ethanol precipitation after phenol-chloroform-isoamylalcohol (25:24:1) extraction. RNAs were then separated on a 6% polyacrylamide gel (19:1) in 1 × TBE buffer containing 7 M urea. RNA was stained with SYBR^TM^ Safe (Invitrogen) diluted 10 000-fold in 1 × TBE buffer for 20 min and visualized by UV epi-illumination. Alternatively, the RNA samples were analyzed through RNA-seq.

### 16S rRNA 3′ end library preparation

To identify RelE1 cleavage site in 16S rRNA 3′ end region, the 3′-OH RNA-seq library was first constructed. RNA was purified from RNA gels after RNase H digestion assay (described above). Gel slices of RNase H treated RNA encompassing the 120–225 nt regions originating from (i) *in vitro* transcription-translation assay, (ii) *in vivo M. tuberculosis* with or without RelE1 overexpression for 4 h or 24 h, (iii) *in vivo M. smegmatis* with or without RelE1 overexpression for 3 h, (iv) *in vitro* 30S subunits incubation assay, and (v) *in vitro* 70S subunits incubation assay were soaked in 300 µl RNA elution buffer (0.3 M NaOAC, 0.1 mM EDTA, 0.1% SDS) overnight to elute RNA from the gels. RNA was precipitated with alcohol and glycogen (20 µg ml^−1^) at −20°C overnight. RNA was dephosphorylated by T4 Polynucleotide Kinase (NEB) and ligated to the adenylated 3p-v4 adaptors. Reverse transcription was performed with Transcriptase inverse SuperScript™ IV using barcode primers. Finally, PCR amplification was performed with primers M.smeg_16SrRNA_RNAseq_3 and A-PE-PCR10 (after five cycles, the program was paused and B_i7RPI2_ACATCG or i7RPI7_ GATCTG was added) using Q5 Polymerase Hot-Start. The library was sequenced by DNBSEQ -G400RS High-throughput Sequencing Set (PE150) in BGI Genomics (Hong Kong). Primers used for the construction of 16S rRNA 3′ end libraries are described in Supplementary File.

## Results

### RelE1 shows severe toxicity in mycobacteria and is inhibited by its cognate RelB1 antitoxin

**Figure 1.**
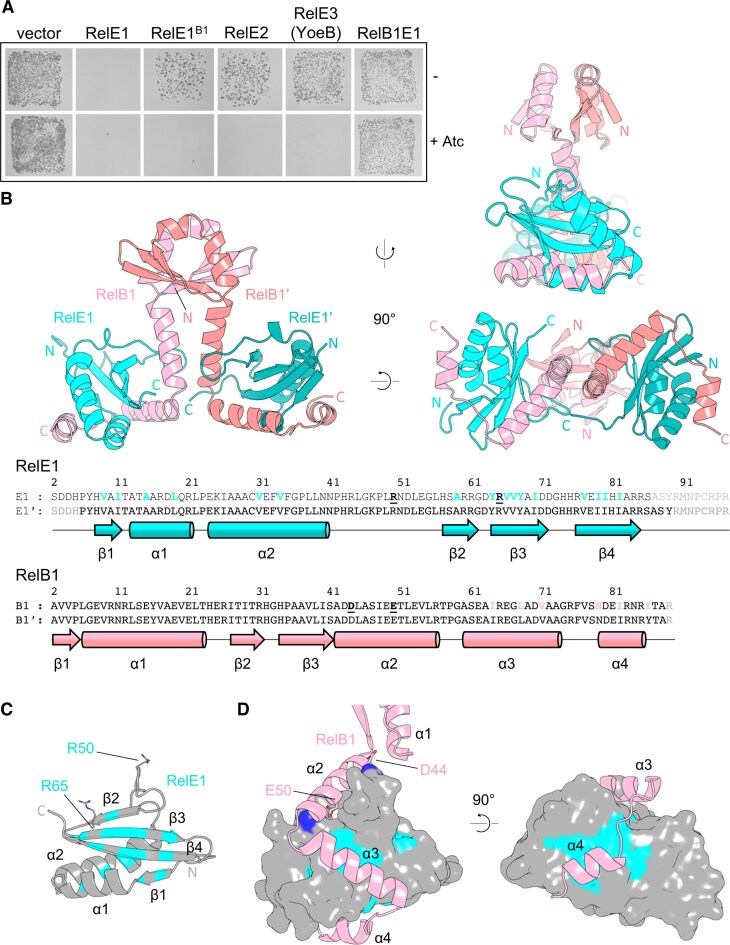
RelE1 is toxic in mycobacteria and is inhibited by its cognate RelB1 antitoxin. (**A**) Expression of RelE toxins *in vivo. Mycobacterium smegmatis* transformed with pGMC vector (−), RelE1, RelE1^B1^, RelE2, RelE3(YoeB), or RelBE1 were plated on LB agar with or without Atc inducer at 100 ng ml^−1^. Plates were incubated for 3 days at 37°C. (**B**) X-ray crystallographic structure of the RelBE1 complex, with 90° views from the side and underneath, shown as cartoons colored cyan or teal for the toxin protomers RelE and RelE′, and pink or salmon for the antitoxin protomers RelB and RelB′. N and C termini are indicated. Secondary structure schematics are displayed alongside full amino acid sequence of each protomer, with cylinders representing α-helices and arrows representing β-strands. Residues shown gray in the amino acid sequence were not resolved within the structure. (**C**) Hydrophobic patches on RelE are highlighted in cyan, and corresponding residues are indicated in panel (B). Residues R50 and R65, identified as forming salt bridges with RelB1, are shown as sticks, and the residues are in bold highlight within panel (B). (**D**) RelB interaction with the surface of RelE. RelB residues D44 and E50 are shown as sticks and the residues are in bold highlight in panel (B); these residues form salt bridges with R50 and R65 of RelE, respectively. RelB1 helices α3 and α4 interact with hydrophobic patches of RelE via corresponding hydrophobic residues, shown as pink residues within panel (B).

To begin investigating the modes of action for *M. tuberculosis* RelE toxins, the genes encoding RelE1, RelE2, and RelE3(YoeB) were amplified and ligated into an integrative plasmid under the control of an anhydrotetracycline inducible promoter and tested for toxicity in *M. smegmatis* (Fig. 1A). While both RelE2 and RelE3(YoeB) show toxicity only in the presence of inducer, we were at first not able to obtain viable transformants with RelE1, even in the absence of inducer. To avoid this problem, we generated a RelE1^B1^ pGMC-based expression plasmid in which RelE1 is under the control of an Atc inducible promoter and the RelB1 antitoxin under the control of the native *relBE1* promoter at a different location on the plasmid (see the ‘Materials and methods’ section). We reasoned that having a basal level of RelB1 upon initial plasmid transformation would allow the obtention of colonies on plates in the absence of inducer. In the presence of Atc inducer, RelE1 overexpression would then be toxic due to an unbalanced expression levels of toxin and antitoxin. Using such a strategy, we were now able to transform RelE1^B1^ plasmid in *M. smegmatis* in the absence of inducer and conditionally induce RelE1 expression and toxicity in the presence of Atc inducer (Fig. 1A). Note that expression of RelE1 and RelB1 TA pair in the context of the *relEB1* operon (RelBE1) did not show toxicity, as expected for a *bona fide* TA system (Fig. 1A). When expressed in *E. coli*, whilst RelE3(YoeB) was again toxic, both RelE1 and RelE2 did not show any detectable toxicity under the same conditions, suggesting that these two toxins could display some host specificity (Supplementary Fig. S1A). We next investigated whether RelE1, RelE2, and RelE3(YoeB) could cleave mRNA *in vivo* when expressed in *M. smegmatis*, using the nEMOTE (nonphosphorylated exact mapping of transcriptome ends) approach recently applied to *M. tuberculosis* HigB1 toxin [18]. The nEMOTE method, which allows the exact mapping of 5′-OH cleavage sites in mRNA *in vivo* on a transcriptome-wide scale, was applied to both *M. smegmatis* wild-type and Δ*rnJ* mutant strains expressing the different toxins as previously described [18]. Of note, the Δ*rnJ* mutant does not produce ribonuclease J and was used to minimize degradation of cleaved toxin targets and thus potentially increase the robustness of the signal of the preferred cleavage sites. Expression of RelE3(YoeB) in both in *M. smegmatis* wild-type and Δ*rnJ* strains led to the identification of multiple cleavage sites in different mRNA targets, with all the cleavages occurring between the second and the third nucleotide within codon sequences (Supplementary Fig. S1B–D; Datasheet 1). In this case, RelE3(YoeB) showed a strong preference for an adenosine just upstream of the cleavage site, similar to what was found for the *E. coli* YoeB toxin [46]. In addition, as recently observed for the *M. tuberculosis* HigB1, the main targets of RelE3(YoeB) include transcripts of genes that are essential in both *M. smegmatis* and *M. tuberculosis* (Supplementary Fig. S1B–D; Datasheet 1). These data strongly suggest that RelE3(YoeB) indeed acts as a ribosome-dependent endoribonuclease [18, 23, 47] with similar activity and motif preference as YoeB [46]. In sharp contrast, nEMOTE analysis of samples taken following expression of RelE1 and RelE2 did not lead to the identification of specific mRNA cleavage when compared to the empty vector (Datasheet 1). This important difference supported the hypothesis that RelE1 and RelE2 differ in their toxic mechanisms from other characterized RelE homologs. Due to its more robust toxicity, we decided to focus on uncovering the structure and the toxic mechanism of RelE1.

### Structure of the RelE1–RelB1 TA complex

Toxin RelE1 and antitoxin RelB1 were co-expressed and purified from *E. coli*, then put into crystallization trials (see the ‘Materials and methods’ section). The resulting hexagonal bifrustum crystals were used to collect X-ray diffraction data. Thirteen datasets were then merged to a final resolution of 2.20 Å. The RelBE1 structure was determined by molecular replacement using the *M. tuberculosis* RelBE2 complex (PDB 3G5O, Miallau *et al.*, 2013) as search model (Fig. 1B and Table 1).

The asymmetric unit contained a heterotetrameric RelB1_2_RelE1_2_ protein complex (Fig. 1B). RelB1_2_RelE1_2_ forms a triangular (or inverted V-shaped) complex with the RelE1 toxin molecules positioned away from each other. Each RelE1 protomer, RelE1 and RelE1′, forms the expected RNase T1-like fold, and the C-terminal region of neither toxin was resolved in the structure (Fig. 1B). Aligning the two RelE1 toxin protomers across all atoms returned a root mean square deviation (RMSD) of 0.390 Å, indicating they are highly similar in fold. The RelB1 antitoxins dimerize through respective domains comprised of an N-terminal three-stranded β-sheet and the α1 helix, which then extends and wraps around cognate RelE1 toxins using helices α2, α3, and α4 (Fig. 1B–D).

Aligning the two RelB1 antitoxins across all atoms returned an RMSD of 0.930 Å, indicating they are also highly similar in fold, but there was slight variation in the position of the N-terminal dimerization domains. The N-terminal domains of RelB1 are similar in fold to DNA-binding regions of antitoxin PhD, suggesting they likely have a role in conditionally cooperative regulation of RelBE1 expression [48]. Interface analysis using PISA [49] identified salt bridges between RelE1 R50 and RelB1 D44, and RelE1 R65 and RelB1 E50, alongside RelE1 hydrophobic patches corresponding to hydrophobic faces of RelB1 α2, α3 and α4 helices, generating complementary interacting surfaces (Fig. 1B–D). ConSURF [50] analysis of amino acid conservation between RelE1 and homologues allowed mapping across the RelB1_2_RelE1_2_ complex (Supplementary Fig. S2). The N-terminal dimerization domain and α2 helix of RelB1 are highly conserved, whilst α3 and α4 amino acids are not conserved (Supplementary Fig. S2). The RelE1 surface residues juxtaposed with RelB1 helix α2 are also highly conserved, but not those surface residues binding RelB1 helices α3 and α4 (Supplementary Fig. S2). Notably, this first RelE1 surface, binding RelB1 helix α2, also includes putatively conserved catalytic residues such as R65. The reduction of conservation for surfaces binding helices α3 and α4 indicates wider variation allowing for specific TA recognition.

Aligning each complex by superposition against one of the RelE toxins allows comparison of overall pose between RelBE complexes (Supplementary Fig. S3). The RelBE1 quaternary structure of course resembled that of the RelBE2 search model (PDB 3G50), but also that of RelBE3(YoeB) (PDB 3OEI, Miallau *et al.*, 2013). In contrast, the RelBE complex from *E. coli* (PDB 4FXE) has a more open antitoxin dimer [51]. When comparing RelBE1 with RelBE from *E. coli* by superposition against one RelE protomer, it is clear there are large variations in the relative TA orientations, despite the overall V-shape of each complex (Supplementary Fig. S3E and F). This indicates a common complex structure specific to the mycobacterial RelBE TA systems.

**Figure 2.**
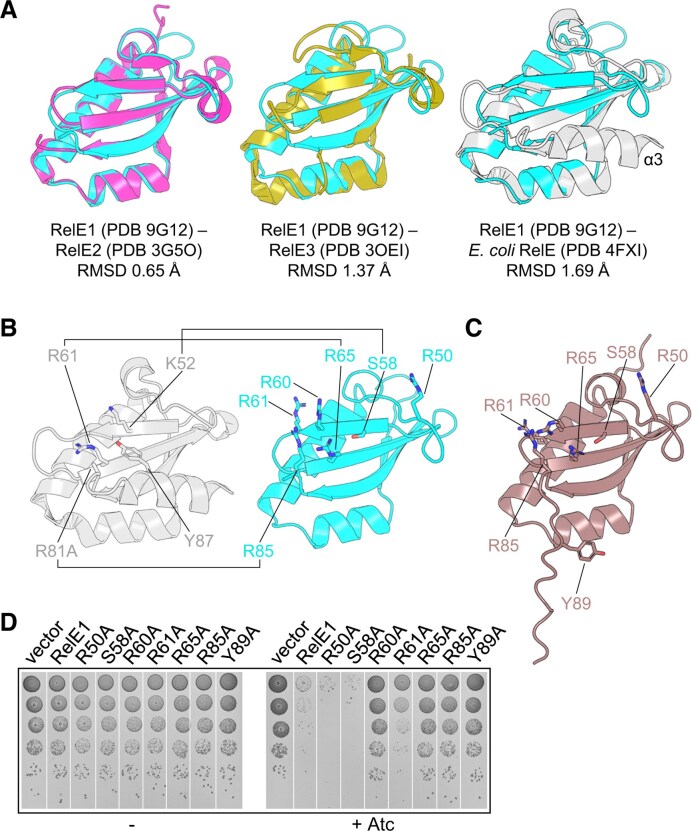
RelE1 shows conservation of residues critical for toxicity. (**A**) Structural superpositions of RelE toxins. *Mycobacterium tuberculosis* RelE1 (PDB 9G12, cyan, this study) was superposed against (i) RelE2 (PDB 3G50, magenta) scoring an RMSD of 0.65 Å across 382 atoms; (ii) RelE3 (PDB 3OEI, olive) scoring an RMSD of 1.38 Å across 352 atoms; and (iii) *E. coli* RelE (PDB 4FXI, gray) scoring an RMSD of 1.69 Å across 363 atoms. PDB 4FXI is the isolated structure of RelE without RelB and has its C-terminal α3 helix resolved in position across the centre of RelE (labeled). (**B**) Catalytic residues K52, R61, and R81 in *E. coli* RelE (gray) correspond in position to residues S58, R65, and R85 in RelE1 (cyan), respectively. Residue Y87 of *E. coli* RelE would correspond to Y89, but this is not resolved in the RelE1 structure, likely because binding to RelB1 causes the C-terminal helix to become disordered. R81 is an R81A mutant in *E. coli* RelE, made in order to reduce toxicity for expression. (**C**) AlphaFold structure of *M. tuberculosis* RelE1 shows residue Y89, but does not fold the C-terminal helix. (**D**) Toxicity of RelE1 derivatives in *M. smegmatis. Mycobacterium smegmatis* transformed with pGMC-vector, RelE1^B1^ or its mutant derivatives (alanine substitution of residues R50, S58, R60, R61, R65, R85, or Y89 within RelE1) were serially diluted, spotted on LB agar plates with or without Atc inducer at 100 ng ml^−1^. Plates were incubated for 3 days at 37°C.

Superpositions of just RelE-like toxins shows high similarity (Fig. 2A). Superposition of RelE1 (this study, PDB 9G12) with RelE2 (PDB 3G5O) gives an RMSD of 0.62 Å (across 569 atoms); RelE1 against RelE3 (PDB 3OEI) gives an RMSD of 1.38 Å (across 439 atoms); RelE1 against isolated RelE from *E. coli* (PDB 4FXI) gives an RMSD of 1.69 Å (across 453 atoms). However, it should be noted that whilst RelE1, RelE2, RelE3, and *E. coli* RelE toxins in complex with cognate RelB antitoxins have unstructured C-terminal regions that are absent in the structures (Fig. 1 and Supplementary Fig. S3), *E. coli* RelE toxin structures derived from RelE alone (PDB 4FXI, Bøggild *et al.*, 2012) or in complex with the ribosome (PDB 4V7K, [52], have a C-terminal helix, α3 (Fig. 2A). Helix α3 of the free RelE toxin therefore appears to be displaced by helix α3 of the cognate RelB antitoxin when forming the complex, such that antitoxicity likely operates both through sequestration and deactivation of the toxin by conformational changes. The position of several known catalytic residues identified on *E. coli* RelE [52] can be mapped onto RelE1 (Fig. 2B). Based on our superpositions, K52, R61, and R81 in *E. coli* RelE likely correspond to S58, R65, and R85 in RelE1, respectively (Fig. 2B). Y87 on helix α3 of *E. coli* RelE would correspond to Y89 of RelE1 and be part of the proposed helix α3, though this region is not present in our structure, and the available AlphaFold database [53] model also plots the C-terminal region as unstructured (Fig. 2C).

Substitutions R50A, S58A, R60A, R61A, R65A, R85A, and Y89A in conserved and/or putative catalytic site residues of RelE1 (Fig. 2B and C) were engineered and mutants were then tested for *in vivo* toxicity in *M. smegmatis* (Fig. 2D). While R50A, S58A, R61A had no, or little impact, the R60A, R65A, R85A, and Y89A substitutions significantly affected RelE1 toxicity, thus highlighting R60, R65, R85, and Y89 as key catalytic residues of RelE1. The RelE1 R65A substitution, which likely corresponds to catalytic residue K95 in HigB1 [18] and R61 in *E. coli* RelE (Fig. 2B) [52] was selected for use as a negative control throughout the study. Of note, the steady state protein expression level analysis performed in *E. coli*, showed a comparable expression between RelE1 and the RelE1 R65A substitution (Supplementary Fig. S4A), as previously observed with the corresponding HigB1 K95A substitution [18]. Yet, in order to confirm the impact of this residue in the native host, we engineered a *M. tuberculosis* Δ*relBE1* knock out strain and showed that indeed, expression of the RelE1 R65A substitution did not inhibit *M. tuberculosis* growth (Supplementary Fig. S4B).

### RelE1 inhibits translation *in vitro* but does not cleave mRNA

**Figure 3.**
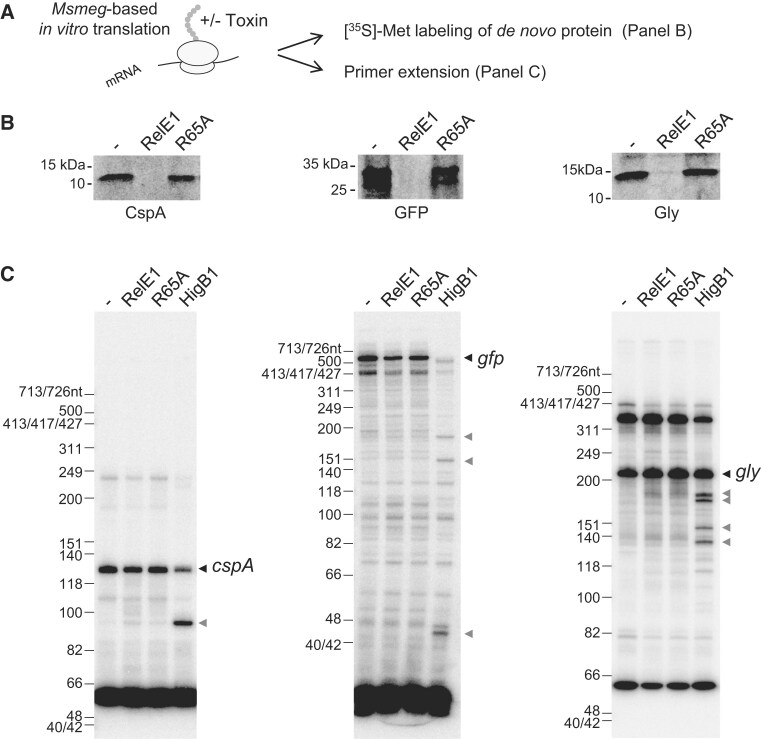
RelE1 inhibits translation *in vitro* but does not cleave mRNA. (**A**) *Mycobacterium smegmatis* (*Msmeg*)-based *in vitro* translation of *cspA, gfp*, and *gly* mRNA performed in absence (−) or presence of RelE1, or RelE1[R65A] was used to monitor the impact of the toxin on protein synthesis (panel B) or on mRNA cleavage (panel C). (**B**) CspA, GFP, and Gly proteins were synthesized for 2 h at 37°C in the presence of [^35^S]-methionine, separated on SDS–PAGE and visualized by phosphorimager. (**C**) *cspA, gfp*, and *gly* mRNA were independently translated for 2 h with or without RelE1 or RelE1 R65A. RNA was extracted and subjected to primer extension. The obtained labeled cDNAs were separated on denaturing urea–polyacrylamide gels and imaged by autoradiography. Uncleaved (black arrows) and cleaved (gray arrows) *cspA, gfp*, and *gly* are indicated. The ribosome-dependent RNase toxin HigB1 was used as positive control under the same conditions.

The structure of RelE1 and the alanine substitution analysis strongly suggests that RelE1 is an RNase. In order to confirm such a hypothesis, RelE1 was purified and first tested for its ability to inhibit translation *in vitro*, using purified *M. smegmatis* ribosomes. We found that purified RelE1 had a strong tendency to aggregate upon freezing. Therefore, we always used freshly prepared RelE1 for activity tests. The results show that RelE1, but not RelE1 R65A, inhibits synthesis of CspA, GFP and Gly model protein products *in vitro*, thus suggesting that RelE1 can prevent translation (Fig. 3A and B). In contrast with the *M. smegmatis*-based *in vitro* translation system, we found that purified RelE1 did not detectably inhibit translation when an *E. coli* transcription/translation system was used (Supplementary Fig. S4C), which is in agreement with the lack of toxicity in *E. coli*. Next, we tested whether RelE1-mediated translation inhibition was due to mRNA cleavage *in vitro* by performing primer extension experiments on total RNA extracts from the *cspA, gfp* and *gly in vitro* translation assays as previously performed with HigB1 [18]. Remarkably, none of the three transcripts were detectably cleaved by RelE1 *in vitro* (Fig. 3C), which is in sharp contrast with the other RelE-like toxins like HigB1 (Fig. 3C) [18, 23] or RelE3/YoeB (Supplementary Fig. S4D). These *in vitro* data are in agreement with the absence of RelE1-dependent mRNA cleavage detected *in vivo* by nEMOTE (Datasheet 1) and suggest that RelE1 inhibits translation by a yet unknown mechanism.

### RelE1 targets the 16S ribosomal RNA directly upstream of the anti-SD region

**Figure 4.**
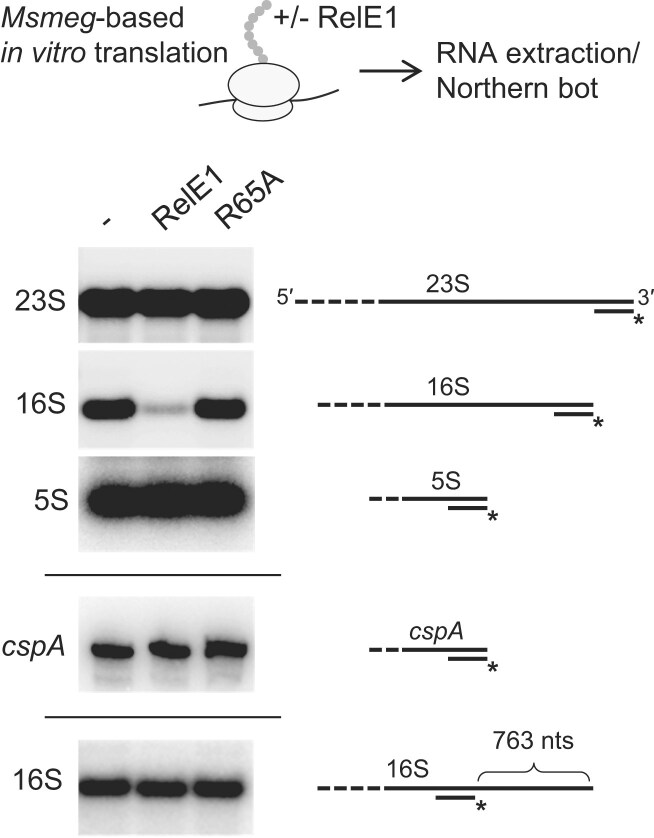
RelE1 specifically targets the 16S ribosomal RNA. Northern blot analysis performed on total RNA extracts obtained following *Msmeg*-based *in vitro* translation of *cspA* in the absence (−) or presence of RelE1, or RelE1 R65A using [^32^P]-labeled 23S, 16S, and 5S rRNA 3′ end probes, *cspA* mRNA 3′ end probe, or 16S rRNA middle probe. RNA was separated on a 1.2% agarose gel and transferred to Hybond-N + membranes. Membranes were hybridized with [^32^P]-labeled oligonucleotide probes at 42°C overnight. The radioactive membranes were exposed to the phosphorimager screens and the signals were imaged using the Typhoon Imager.

**Figure 5.**
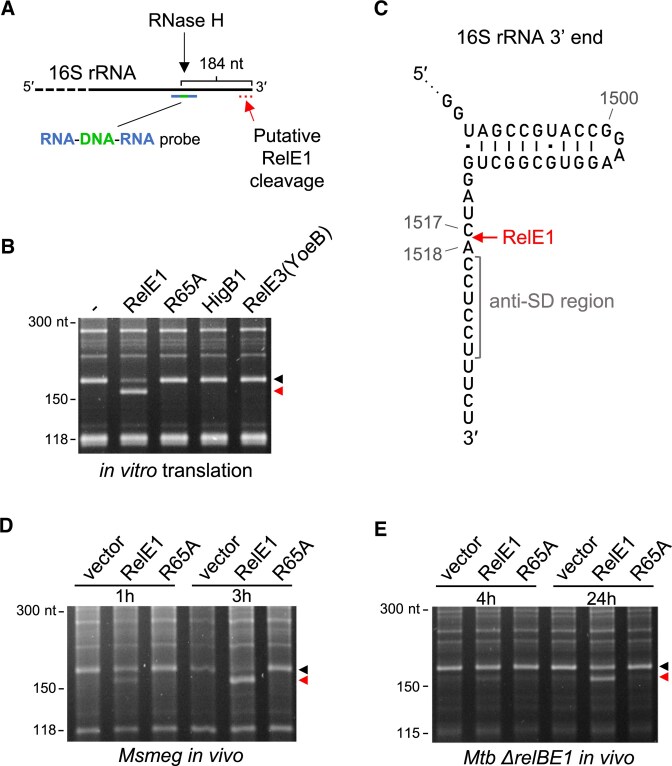
RelE1 cleavage occurs directly upstream of the anti-SD region in the 16S rRNA 3′ end. (**A**) Strategy used to identify the 3′ end cleavage of the 16S rRNA. A DNA–RNA–DNA hybrid probe (blue and green color) positioned at 184 nt from the 3′ end of the 16S rRNA was used to promote RNase H cleavage and generate a 16S rRNA fragments that can be separated and sequenced. (**B**) RelE1, but not RelE1 R65A, HigB1, or RelE3(YoeB), cleaves the extreme 3′ end of the 16S rRNA *in vitro*. RNAs extracted from the *M. smegmatis*-based *in vitro* translation of *cspA* mRNA in the absence (−) or presence of RelE1, RelE1 R65A, HigB1, or YoeB were subjected to an RNase H treatment in the presence of hybrid probe, separated on a denaturing urea–polyacrylamide gel and stained by SYBR-safe. The black arrow indicates the expected wild-type 16S rRNA 3′ end RNA fragment and the red triangle indicates the RelE1-cleaved 16S rRNA fragment. (**C**) Position of the RelE1 cleavage site (red arrow) within the 16S rRNA 3′ end region identified following 3′ end sequencing of the cleaved fragment. (**D**) RelE1 cleaves the 16S rRNA *in vivo*. RelE1 or RelE1 R65A was expressed from a pGMC-based vector for 1 h or 3 h in *M. smegmatis*, and (**E**) for 4 h or 24 h in *M. tuberculosis ΔrelBE1* mutant in the presence of Atc inducer. Total RNA was extracted and subjected to a RNase H treatment, separated and stained as described as described in panel (A).

We next asked whether RelE1 could specifically target ribosomal RNA instead of mRNA. To achieve this aim, total RNA from *in vitro* translation inhibition experiments obtained in the presence of RelE1 or RelE1 R65A was extracted and northern blots were first performed using probes located at the 3′ ends of the *M. smegmatis* 16S and 23S rRNA (Fig. 4). Strikingly, a severe decrease in the 16S rRNA level was observed in the presence of RelE1, while the RelE1 R65A inactive control had no effect (Fig. 4). In addition, no RelE1 cleavage could be detected in 23S and 5S rRNA, or in a *cspA* mRNA control. These data strongly suggest that RelE1 inhibits translation by specifically cleaving the 16S rRNA. Remarkably, when a probe located in the middle part of 16S rRNA was used, no decrease in the size and intensity of the 16S rRNA band could be observed (Fig. 4), indicating that rRNA cleavage by RelE1 occurs very close to the 3′ end region of the 16S rRNA. To further investigate RelE1 cleavage in the 16S rRNA, we applied an RNase H-based method that allows the visualization of short differences in size between RNA fragments following separation on a urea acrylamide gel. In this case, an RNA/DNA/RNA hybrid probe that specifically recognizes the 16S rRNA of *M. smegmatis* at 184 nucleotides from its 3′ end was developed and used (Fig. 5A). Following RNase H treatment, a fragment of about 184 nucleotides is thus produced either in the absence of toxin or in the presence of the RelE1 R65A inactive toxin (Fig. 5B). In sharp contrast, a shorter rRNA fragment was observed in the presence of RelE1, indicating that RelE1 indeed cleaves several nucleotides before the 3′ end of the 16S rRNA (Fig. 5B). Noticeably, the two other RelE-like toxins HigB1 and RelE3/YoeB that cleave mRNA did not produce such a cleavage of the 16S rRNA (Fig. 5B). Further 3′-OH sequencing of the 16S rRNA fragments precisely identified RelE1-dependent cleavage between nucleotides C1517 and A1518 of 16S rRNA, corresponding to one nucleotide before the anti-SD sequence (Fig. 5C). Note that RelE1 cleavage, which occurs only 11 nucleotides from the 16S 3′ end, generates a rRNA fragment that is too short to be detected by nEMOTE [43].

We next asked whether RelE1 expression would lead to similar cleavage *in vivo*. In this case, RelE1 (or RelE1 R65A) was expressed for 1 and 3 h (corresponding to one doubling time) in *M. smegmatis*, or 4 and 24 h (corresponding to one doubling time) in *M. tuberculosis*. Total RNA was extracted and subjected to RNase H treatment, as performed *in vitro*. In agreement with our *in vitro* data, RelE1 also cleaved the 16S rRNA between C1517 and A1518 *in vivo* in both bacteria (Fig. 5D and E). Remarkably, distinct 16S rRNA cleavage by RelE1 was already detected after short induction times and increased significantly after a prolonged expression corresponding to a doubling time for each bacterium (Fig. 5D and E).

Together these data indicate that the 16S rRNA is the main target of RelE1 and that cleavage by the toxin leads to the formation of ribosomes deprived of a functional anti-SD region, likely reflecting the acute toxicity of RelE1 *in vivo*. The fact that RelE2 is structurally very similar to RelE1 [20] and that both toxins do not cleave mRNA *in vivo* in our nEMOTE analysis suggests that a similar toxic mechanism might be employed by the two toxins. Accordingly, expression of RelE2 also induces a specific cleavage of the 16S 3ʹ-end, although less pronounced than RelE1, which is also more toxic (Fig. 1A and Supplementary Fig. S4E).

### RelE1 cleavage does not require ongoing translation

**Figure 6.**
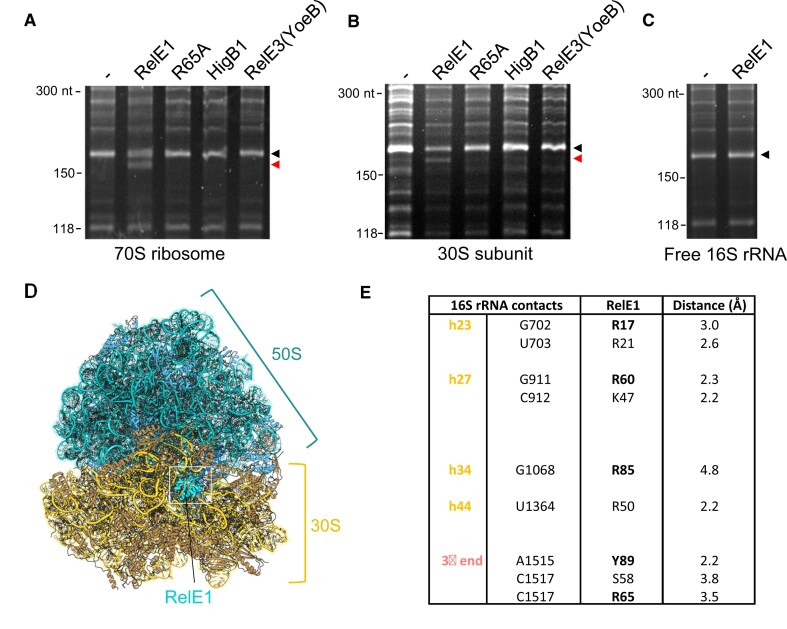
RelE1 cleavage does not require active translation. Purified *M. smegmatis* 70S ribosomes (**A**), 30S subunits (**B**), or 16S rRNA (**C**) were incubated with the indicated toxin at 37°C for 2 h. RNA was extracted, subjected to an RNase H treatment, separated and stained as described in Fig. 5. The black arrow indicates the expected wild-type 16S rRNA 3′ end RNA fragment and the red arrow indicates the RelE1-cleaved RNA fragment. (**D**) Molecular docking of RelE1 with *M. smegmatis* 70S ribosome showing RelE1 docked to the 30S subunit. Ribosomal RNA and proteins of the 50S colored teal and blue, respectively, and ribosomal RNA and proteins of the 30S colored gold and yellow, respectively, and RelE1 colored cyan. The structures of RelE1 and the *M. smegmatis* ribosomal 30S subunit were extracted from the RelBE1 crystal structure (PDB 9G12) and the full *M. smegmatis* ribosome (PDB 5O61), then uploaded to HADDOCK for docking. Docking was performed using standard parameters, selecting RelE1 residues R17, R21, R60, R65, R85, and Y89, and the final 3′ 16S rRNA bases as target residues. (**E**) Table summarising the residues of RelE1 predicted to interact with the 16S rRNA.

Although our data showed that RelE1 inhibits translation most likely by targeting the 16S rRNA, it remained unclear as to whether such deleterious activity would require active translation as is the case for ribosome-dependent mRNA interferase toxins like HigB, YoeB, or RelE [18, 47, 52]. In order to test this, we purified both *M. smegmatis* 70S and 30S ribosomal fractions and incubated them separately with purified RelE1 or RelE1 R65A, extracted RNA and performed RNase H treatment (Fig. 6A and B). The data show that RelE1, but not RelE1 R65A, can cleave the 16S rRNA from both the 70S ribosome and the purified 30S subunit, indicating that RelE1 does not need ongoing translation to be active. This is in sharp contrast with other RelE toxins (Mansour *et al.*, 2022). In addition, the fact that free 16S rRNA was recalcitrant to RelE1 (Fig. 6C) strongly suggests that RelE1 cleavage of the 16S rRNA 3′ end relies on a fully assembled 30S subunit.

The fact that RelE1 can directly target the anti-SD sequence to inhibit translation suggests that there might be sufficient space in the 30S, free or engaged with the 50S, to accommodate a toxin in the vicinity of the extreme 3ʹ end of the 16S rRNA. In order to first investigate whether RelE1 could bind in the vicinity of the anti-SD region of the 16S rRNA we performed molecular docking experiments using HADDOCK [54]. We extracted RelE1 from the RelBE1 complex (PDB 9G12) and the 30S subunit from the solved structure of the *M. smegmatis* 70S ribosome (PDB 5O61) for docking. The 30S was used instead of the full 70S because otherwise the computational job would not run, presumably due to the size. The resulting proposed and preliminary docking model depicts RelE1 bound to the unoccupied pocket adjacent to the terminal 3′ bases of the 16S rRNA within the 30S subunit (Fig. 6D). As PDB 5O61 does not contain residues of the anti-SD sequence due to lack of density, this docking pose represents what might be a ‘post-cleavage’ state of the ribosome, though we note again this is currently only a model. Within this model, R17, R21, K47, R50, R60, and R85 can be loosely predicted as interacting with the sugar-phosphate backbone of 16S rRNA (Supplementary Fig. S5A and B, and Fig. 6E), suggesting potential roles in RNA binding. In support of this, alanine substitution of R17, R60, R85 or Y89 affects RelE1 toxicity (Fig. 2D and Supplementary Fig. S5C). Similarly, alanine substitution of R65 impacts RelE1 toxicity (Fig. 2D), and this residue has the potential to form interactions with the incoming 3′ 16S rRNA bases in our model (Supplementary Fig. S5A and B; Fig. 6E). Coupled with our toxicity data, this predictive model demonstrates accessibility of the 16S rRNA 3′ end to RelE1, supports R60, R65, R85, and Y89 as catalytic residues, and offers potential insight as to the requirement for other rRNA-interacting residues in RelE1 toxicity *in vivo*.

## Discussion

This work reveals an unexpected toxic mechanism involving the specific removal of the anti-SD region of the 30S ribosomal subunit by a RelE-like toxin. We show that in sharp contrast with established RelE toxins, the RelE1 RNase toxin of *M. tuberculosis* does not cleave mRNA during translation but instead specifically targets the small ribosomal subunit, cleaving the exposed 3′ end region of the 16S rRNA between C1517 and A1518, just upstream of the anti-SD region. As a consequence, accumulation of non-functional 30S subunits may lead to a severe drop in translation initiation and to the subsequent inhibition of bacterial growth (and eventually cell death). Our toxicity data are supported by a predictive model that demonstrates both accessibility of the 16S rRNA 3′ end region to RelE1 and complementary interactions between RelE1 and 16S rRNA that would facilitate such a novel toxic mechanism. We do note, however, that the α3 helix of RelE1, needed for toxicity of *E. coli* RelE (Fig. 2B), is not folded within our X-ray structure, nor in the AlphaFold model. The modeled position of catalytic residue Y89 should therefore be considered cautiously until a time when an experimentally derived structure of the RelE1:30S complex can be obtained.

Overall, RelE1 shares high structural similarity with RelE (RMSD = 1.80 Å across 370 atoms), YoeB (RMSD = 1.84 Å across 374 atoms), and HigB1 (RMSD = 1.53 Å across 307 atoms). However, in stark contrast to each of these related toxins, RelE1 lacks mRNase activity and does not require ongoing translation for activity [18, 55, 56]. Furthermore, RelE1 does not bind to the ribosomal A-site adjacent to the decoding centre but likely occupies a small pocket located toward the 3′ end of 16S rRNA. For each of the RelE, YoeB, and HigB1 toxins, an extensive network of contacts has been mapped between respective mRNases and 16S rRNA helices h18, h30/31, h34, and h44, supported by additional interactions with bound mRNA substrates (Supplementary Fig. S6) [18, 52, 57]. Superposing each of these toxins onto RelE1 highlights an absence of several RNA-interacting residues; none of the HigB1 or RelE amino acids involved in recognition of h34 have structurally equivalent residues in RelE1 (Supplementary Figs S6 and S7A and B). Additionally, RelE1 contains fewer decoding centre contacts than HigB1, and is completely devoid of h18 contacts otherwise present in RelE. In contrast, there does appear to be a moderate degree of structural conservation for h30–31 contacts from RelE and HigB1; RelE1 R17/R21 correspond to RelE K13/K17 and HigB1 K27/K31, respectively. Residues involved in binding to the decoding centre bases of h44 are also universally conserved between all four toxins, and mRNA-interacting residues similarly show high structural conservation between respective homologs. The absence of RNA-interacting residues is most apparent when compared against YoeB. Of the six basic amino acids shown to contact h18, h30, and h31, only two are structurally conserved in RelE1 (Supplementary Fig. S7C; K25 and R61). YoeB binds to the ribosomal A-site as a dimer, thereby occupying a significantly larger area that in turn allows for additional contacts to form with h34 in the 30S head domain [57]. Currently, we lack structural evidence for the existence of a RelE1 dimer in solution, and so the inability of the toxin to bind to the A-site may stem from a comparably lower occupancy relative to other mRNase toxins. Given these toxins share the same overall RNase T1-like fold, we hypothesize that the conservation of mRNA recognition motifs between mRNases and RelE1 merely reflects shared catalytic functionality, and not a common biological target or mode of action.

RelE toxins have previously been shown to function in a similar catalytic manner to RNase T1, with phosphodiesterase activity reliant on proton abstraction by a general base. This in turn activates the 2′ OH of RNA for nucleophilic attack on the scissile phosphate, with protonation of the leaving group mediated by a general acid [58]. To discern the role of conserved RelE1 residues in catalysis, we compared structurally conserved amino acids from each ribosome-bound toxin structure to predict the general acid and base residues of RelE1 (Supplementary Fig. S7D–F). RelE amino acids R45, K52, K54, R61, R81, and Y87 all recognise mRNA within the A-site (Supplementary Fig. S7D), with the latter three residues all essential for RelE cleavage activity *in vitro* [52]. Each of these amino acids likely corresponds to RelE1 R50, S58, R60, R65, R85, and Y89 respectively, with loss-of-function RelE R61A, R81A, and Y87A mutations synonymous with non-toxic RelE1 R65A, R85A, and Y89A substitutions (Fig. 2D). RelE K52 functions as the general base, whilst R81 is the general acid responsible for protonating the scissile phosphate leaving group. Based on our structural alignments, RelE1 R85 and S58 would therefore constitute the general acid-base pair respectively, whilst Y89, R60, and R65 would interact with the target substrate. Although the RelE1 S58A mutation had no impact on toxicity (Fig. 2D), the equivalent RelE K52 residue was also dispensable for mRNA cleavage activity *in vitro* [52], perhaps owing to the surrounding cluster of basic residues compensating for the absence of the canonical general base. The highly electropositive microenvironment surrounding S58 may lower the p*K*_a_ of the side-chain hydroxyl to assist base catalysis. Alternatively, phosphorylation of S58 might activate this amino acid as the general base, though there is no evidence for the existence for RelE1 phosphopeptides in the literature, suggesting phosphorylation is not a key regulator of RelE1 catalysis[59]. Similar trends are observed when comparing the essentiality of RelE1 amino acids against known mRNA-interacting residues from YoeB and HigB1. Specifically, HigB1 K58, E66, R68, R77, and K95 possibly correspond to RelE1 K47, S58, R61, R65, and R85, respectively (Supplementary Fig. S7E). Essential YoeB residues E46, R65, H83, and Y84 likely also correspond to RelE1 K47, R65, R85, and Y89, respectively, with the α3 helix of either toxin remaining unresolved in their respective structures (Supplementary Fig. S7F). Catalytic studies probing the mechanism of YoeB cleavage invoked H83 as the general acid [47, 60], which corresponds to RelE1 R85, once again supporting the role of R85 as the general acid of RelE1. In contrast, the putative general base S58 now aligns with YoeB S57, which has no known role in catalysis. Instead, the general base of YoeB E46 co-localises with RelE1 K47. Although no studies probing the catalytic mechanism of the HigB1 toxin have been reported, basic HigB1 amino acids K95 and K58 likely correspond to putative general acid-base RelE1 residues R85 and K47. This may also hint at these specific residues playing an important role in HigB1 activity. Accordingly, mutation of either residue in HigB1 resulted in a complete loss of cellular toxicity [18], confirming their importance for mRNase cleavage. Collectively, these structural alignments clarify the role of the conserved RNase T1-like fold shared within Rel superfamily toxins, though the precise roles of key RelE1 amino acids in catalysis requires experimental validation.

Our data also suggest that targeting 16S rRNA is a mechanism that is possibly shared with RelE2. So far, the different phenotypes associated with RelE1 in *M. tuberculosis* are related to its overexpression and the single deletion of *relE1* did not lead to any significant phenotype [20, 61]. Yet, the fact that RelBE1 and RelBE2 are structurally very similar and share the same cellular target suggests that they could have redundant functions that might be detected only in the absence of both TA systems. Whether other RelE-like homologs from bacteria or archaea have a similar target, and how one can discriminate between RelE homologs targeting mRNAs vs those targeting 16S rRNA, remains to be investigated [62].

Several unrelated toxins were previously shown to target rRNA, although in most cases rRNA did not appear to be their sole target [63–68]. The *E. coli* endoribonuclease toxin MazF was shown to specifically cleave the 16S rRNA *in vitro* after position A1500 and to a lesser extend after C1396 [63], but a later transcriptome-wide analysis demonstrated that MazF mainly targets mRNA *in vivo* [69]. Expression of the *M. tuberculosis* MazF6 toxin also revealed cleavage sites in both 16S and 23S in *E. coli* rRNA [64]. In this case, the identified 16S cleavage site occurred within the anti-SD sequence of the *E. coli* ribosome, after position U1537, corresponding to four nucleotides downstream of the RelE1 cleavage site identified in this work. Yet, as described for the *E. coli* MazF, MazF6 additionally targeted many mRNA transcripts [64] and it remains to be determined whether such rRNA cleavage is relevant in mycobacteria. The MazF3 toxin of *M. tuberculosis* was shown to cleave the 23S rRNA in the evolutionarily conserved helix/loop 70 both *in vivo* and *in vitro* in *E. coli* and *M. smegmatis*, and *in vitro* with purified *M. tuberculosis* 23S rRNA [65]. Noticeably, MazF3 did not cleave the 70S ribosome, suggesting that it only targets the free 50S subunit [65]. However, as observed with MazF6, multiple mRNA targets were also identified in the case of MazF3.

Certain toxins have been shown to preferentially or solely target rRNA. In the case of the MazF11 toxin of *M. tuberculosis* it was recently shown that the toxin preferentially cleaves the 3′ end of 16S rRNA *in vivo* in *M. smegmatis*, at the same position as RelE1. Although both MazF11 and RelE1 are structurally unrelated, this further highlights the anti-SD region as a hot spot for poisonous toxins[70]. The PIN domain containing RNase toxins VapC20 and VapC26 of *M. tuberculosis* were shown to specifically cleave after position G2661 within the Sarcin Ricin loop of the 23S rRNA, at the same conserved position where Sarcin and other fungal ribotoxins cleave rRNA to inhibit translation and induce cell death by apoptosis [67, 68]. The bacterial rRNase Colicin E3, a plasmid-encoded cytotoxic translation inhibitor produced during certain stress to kill rival bacteria was shown to specifically cleave the 16S rRNA after position A1493 in the vicinity of the ribosomal A-site [71], 41 nucleotides upstream of the RelE1 cleavage site. In this case, Colicin E3 cleavage at the ribosomal decoding center was proposed to prevent the access of tRNA and ribosomal factors, leading to cell death. Although the regions of the 16S rRNA that are targeted by Colicin E3 and RelE1 are different, rRNase has deleterious consequences for the cell, thus further emphasizing the advantage of selectively targeting the 16S rRNA by poisonous toxins.

Removal of the 16S rRNA 3ʹ-end region by RelE1 may exert a dramatic effect on translation initiation. Accordingly, RelE1 expression for a duration corresponding to only one doubling time was sufficient to induce the cleavage most of the total 16S 3ʹ-end. Although the conditions leading to RelE1 activation *in vivo* are currently not known, the fact that RelE1 can target free 30S subunits as well as 70S ribosomes independently of ongoing translation is in agreement with the acute toxicity of RelE1 and suggests an irreversible collapse of translation upon toxin induction. In addition, we cannot exclude that pre-30S ribosomal particles might also be targeted by the toxins, thus potentially aborting ribosome biogenesis [64]. Noticeably, previous work performed in *E. coli* showed that starvation stress induces the formation of a significant number of free ribosomal subunits that are efficiently degraded by endogenous RNases [72, 73]. In this case, degradation of the 30S initiates with the removal of about 20 nucleotides at the extreme 3ʹ-end of the 16S rRNA by the exonuclease RNase PH, which is subsequently followed by a rapid degradation cascade mediated by other RNases, including RNase E, RNase II, and RNase R [72, 74]. Considering the fact that RelE1 cleavage occurs in close proximity to RNase PH cleavage (i.e. RelE1 cleavage removes 11 nucleotides), we hypothesize that initial cleavage by RelE1 *in vivo* could possibly trigger a similar degradation cascade involving other endogenous RNases of *M. tuberculosis*.

## Supplementary Material

gkaf1070_Supplemental_Files

## Data Availability

The crystal structure of RelBE1 has been deposited in the Protein Data Bank under accession number 9G12. The nEMOTE seq data generated in this study have been deposited at the European Nucleotide Archive database under accession code PRJEB90910. All other data needed to evaluate the conclusions in the paper are present in the paper and/or Supplementary Data.

## Notes

Present address: Max Planck Unit for the Science of Pathogens (MPUSP), 10117, Berlin, Germany
