# Supplementary material for: Ribonuclease toxin RelE1 inhibits growth of Mycobacterium tuberculosis through specific cleavage of the ribosomal anti-Shine–Dalgarno region
Source: Nucleic Acids Res. 2025 Nov 17;53(21):gkaf1070. doi: 10.1093/nar/gkaf1070 (PMC12620021; doi:10.1093/nar/gkaf1070)
Supplement: gkaf1070_Supplemental_Files [file gkaf1070_supplemental_files.zip › HAN et al Supplementary File.pdf]

SUPPLEMENTARY FILE

Ribonuclease toxin RelE1 inhibits growth of *Mycobacterium tuberculosis* through specific cleavage of the ribosomal anti-Shine-Dalgarno region

Xue Han, Izaak N. Beck, Moise Mansour, Tom J. Arrowsmith, Roland Barriot, Paul Chansigaud, Carine Pagès, Hussein Hamze, Hatice Akarsu, Laurent Falquet, Peter Redder, Xibing Xu, Tim R. Blower, Pierre Genevaux

This file contains seven supplementary Figures and one Table of primers used in this study.

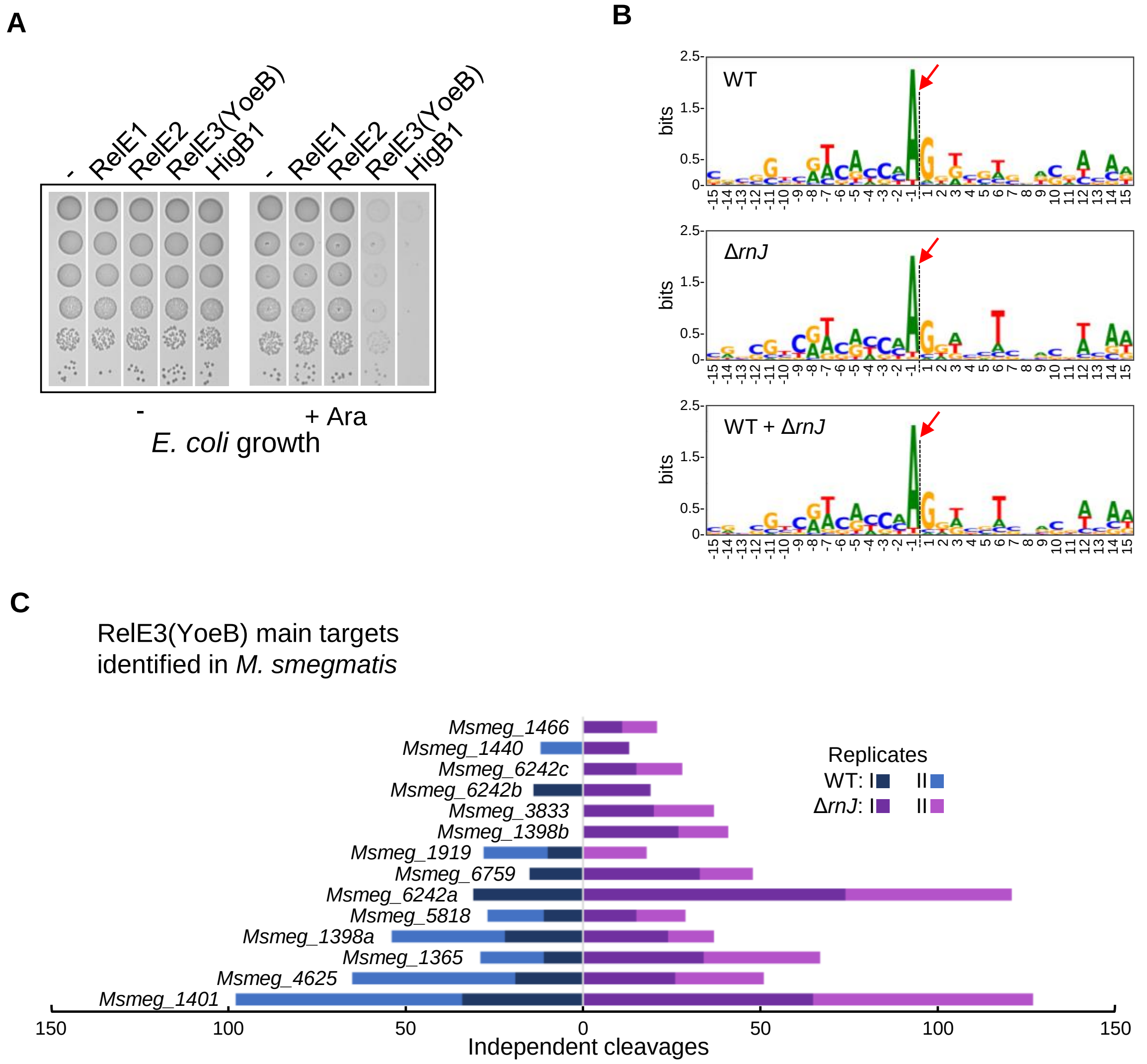

D

Genes	position (codon)	motif	genomic position	Cleavages /replicates	*Mtb* ortholog	Essential	Function
MSMEG_1365	79/131	AA/G	+1459501	96/4	*Rv0652*	yes	50S ribosomal protein L7/L12
MSMEG_1398	105/125	CA/G	+1498616	91/4	*Rv0682*	yes	30S ribosomal protein S12
MSMEG_6759	141/506	CA/G	+6804185	63/3	*Rv3696c*	no	glycerol kinase
MSMEG_5818	392/405	GT/G	+5884998	56/4	*Rv0589*	no	virulence factor Mce
MSMEG_1919	29/85	CA/G	-2000311	46/3	*Rv3219*	no	transcription factor WhiB
MSMEG_1398	75/125	CA/G	+1498526	41/2	*Rv0682*	yes	30S ribosomal protein S12
MSMEG_3833	72/480	CA/C	-3904604	37/2	*Rv1630*	yes	30S ribosomal protein S1
MSMEG_6242	29/424	CA/C	-6308717	28/2	no	no	alcohol dehydrogenase
MSMEG_6242	42/424	AA/G	-6308678	33/2	no	no	alcohol dehydrogenase
MSMEG_1440	14/94	CA/T	+1540122	25/2	*Rv0705*	yes	30S ribosomal protein S19
MSMEG_1466	106/106	TG/A	+1562625	21/2	*Rv0715*	yes	50S ribosomal protein L24
MSMEG_1401	34/397	AA/G	+1501514	225/4	*Rv0685*	yes	elongation factor Tu
MSMEG_6242	37/424	AA/G	-6308693	152/3	no	no	alcohol dehydrogenase
MSMEG_4625	104/104	TA/G	-4709385	116/4	*Rv2442c*	yes	50S ribosomal protein L21

Supplementary Fig. 1: Toxicity of RelE-like toxins in *E. coli* and nEMOTE analysis of RelE3(YoeB) toxin targets and recognition motif. (**A**) Both RelE1 and RelE2 are not detectably toxic in *E. coli*. Cultures of *E. coli* DLT strain transformed with either pK6-RelE1, RelE2, RelE3/YoeB or HigB1 were serial diluted and spotted on LB Km plates in the presence or absence of 0.2% arabinose inducer. Plates were incubated

overnight at 37 °C. (**B**) Logoplots showing RelE3(YoeB) preferred motif generated from multiple cleavage sequences associated with cleavage events obtained for the WT (n=545), the Δ*rnJ* mutant (n=848) or both WT+Δ*rnJ* (n=1393). The x axis represents the 15 nucleotides upstream and downstream of the cleavage site that is located by the position between −1 and 1 (red arrow and dash line), and the default label for the y axis is bits. (**C**) Main RelE3(YoeB) mRNA targets identified *in vivo* using nEMOTE after 3 h expression in *M. smegmatis* WT (on the left; shade of blue; replicates I and II) and Δ*rnJ* (on the right; shade of violet; replicates I, and II). Bar length represents the number of independently observed cleavage events for each unique target site (x axis). The name of the gene of the cleaved mRNA is given on the left. (**D**) General information about the cleavage sites identified in this work. The position of the cleaved codons is given as codon position per total number of codons of the transcript. The cleaved codon sequence is indicated as "motif" and the bar slash shows where cleavage occurs. The position of the first nucleotide after cleavage in *M. smegmatis* mc^{2}155 genome is given in the "genomic position" column. The number of total detected cleaved RNA molecules (cleavages) for each transcript and the number of replicates in which such cleavages were identified (replicates) are shown. The presence of the targeted genes in *M. tuberculosis* (*Mtb* ortholog) and the essentiality of such genes in *M. tuberculosis* (Essentiality) are also indicated. Functional annotation of conserved genes is based on information available at UniProtKB.

A

B

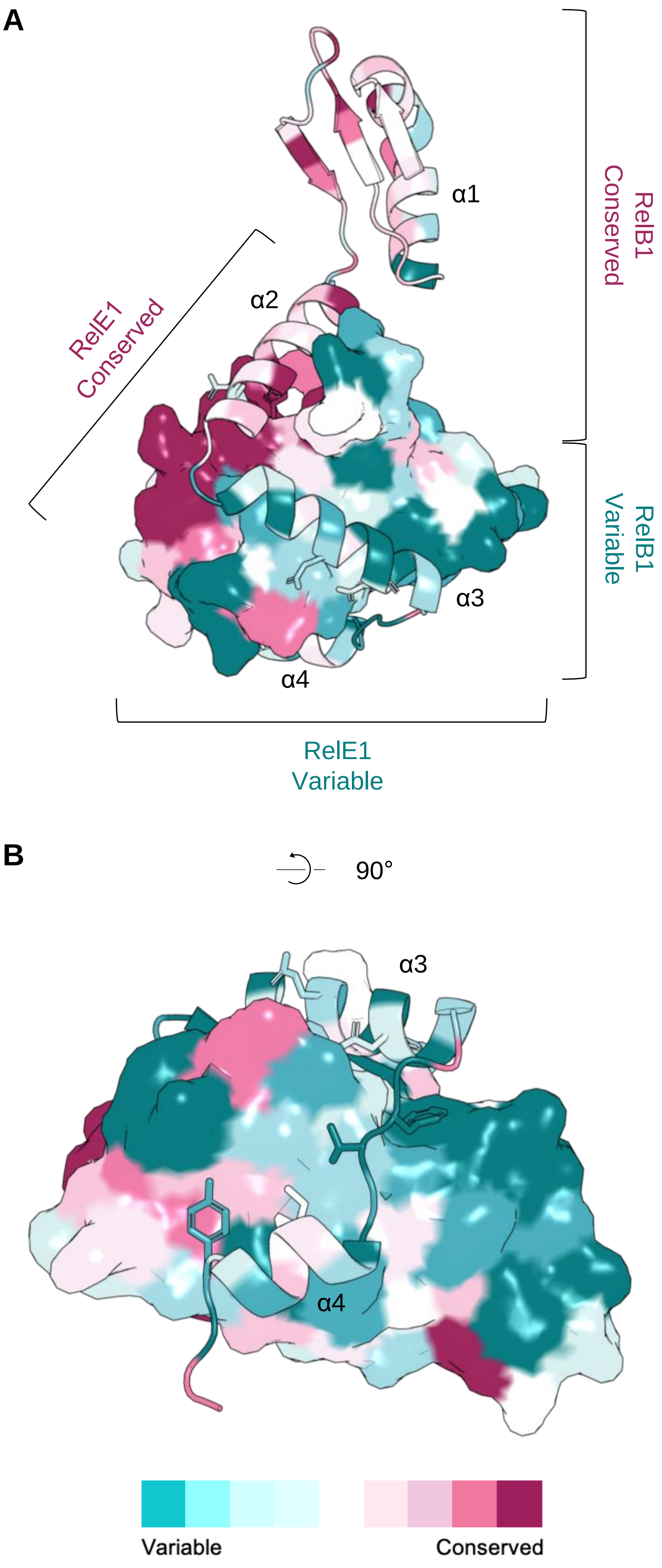

Supplementary Fig. 2: Conservation of RelBE1 surface residues. (**A**) Cartoon surface rendered RelE1 dimerized with RelB1. Brackets indicate levels of conservation for both RelE1 and RelB1. (**B**) Rotated and enlarged view of (A). Secondary structure

labels for RelB1 α-helices are included throughout. Cartoon and surface representations of the RelBE1 dimer from the RelBE1 crystal structure are rendered in ConSurf coloring scheme, shown as a gradient below (B), to represent amino acid conservation.

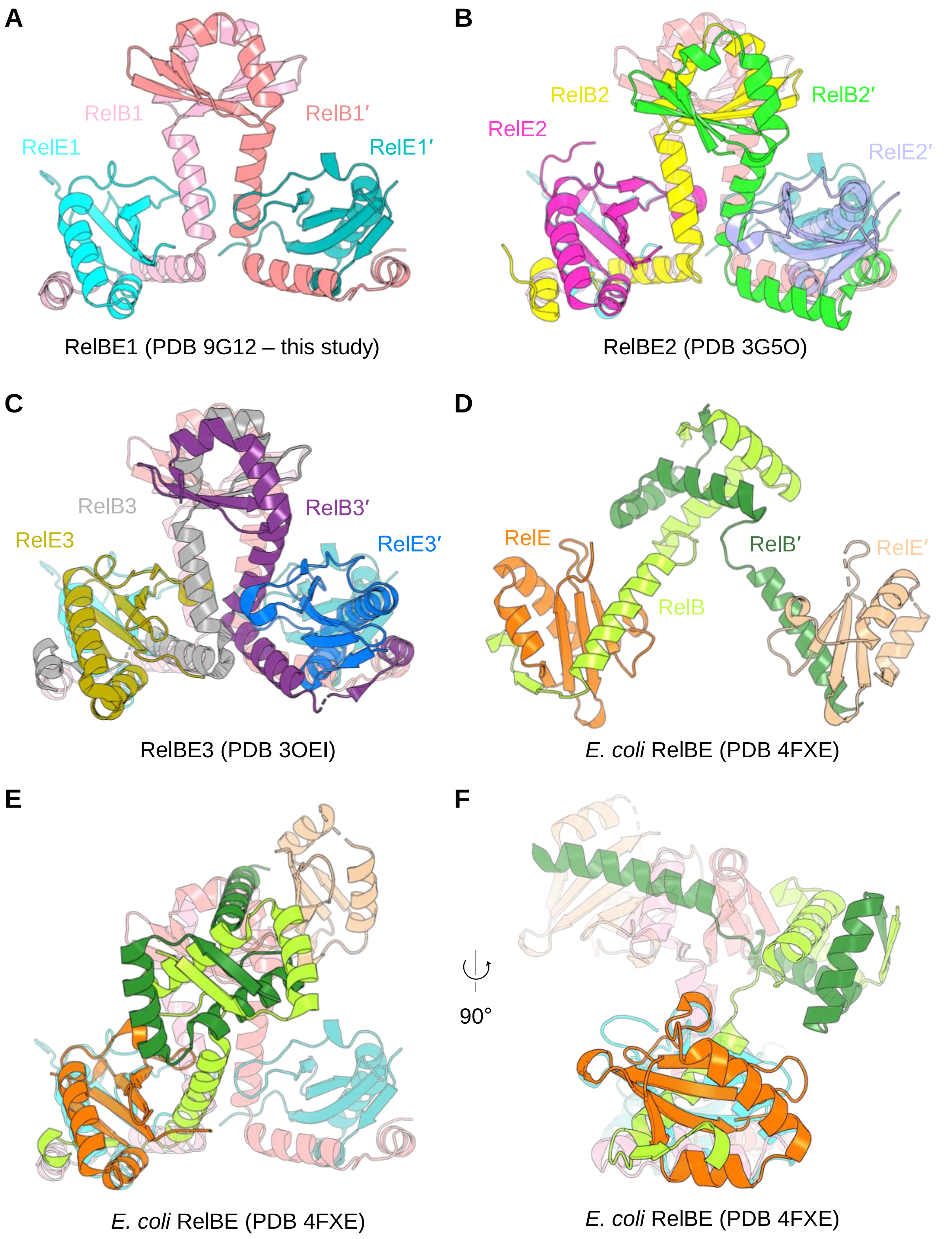

Supplementary Fig. 3: Structural comparison of RelBE complexes. (**A**) Cartoon representation of the RelBE1 complex from this study (PDB 9G12), same pose and coloring as Fig. 1B. (**B**) RelBE2 complex (PDB 3G5O) superposed specifically against the RelE1 toxin, rather than between the two complexes as whole entities. This shows

that variations across the complexes. (**C**) RelBE3 complex (PDB 3OEI) superposed also specifically against the RelE1 toxin. (**D**) RelBE complex from *E. coli* (PDB 4FXE), oriented similarly to RelBE1 in (A). (**E**) RelBE complex from *E. coli* (PDB 4FXE) superposed specifically against the RelE1 toxin within the RelBE1 complex, to show the huge variation in complex positioning between the *M. tuberculosis* RelBE1, RelBE2 and RelBE3 complexes and that from *E. coli*. (**F**) A second view of complexes in (E).

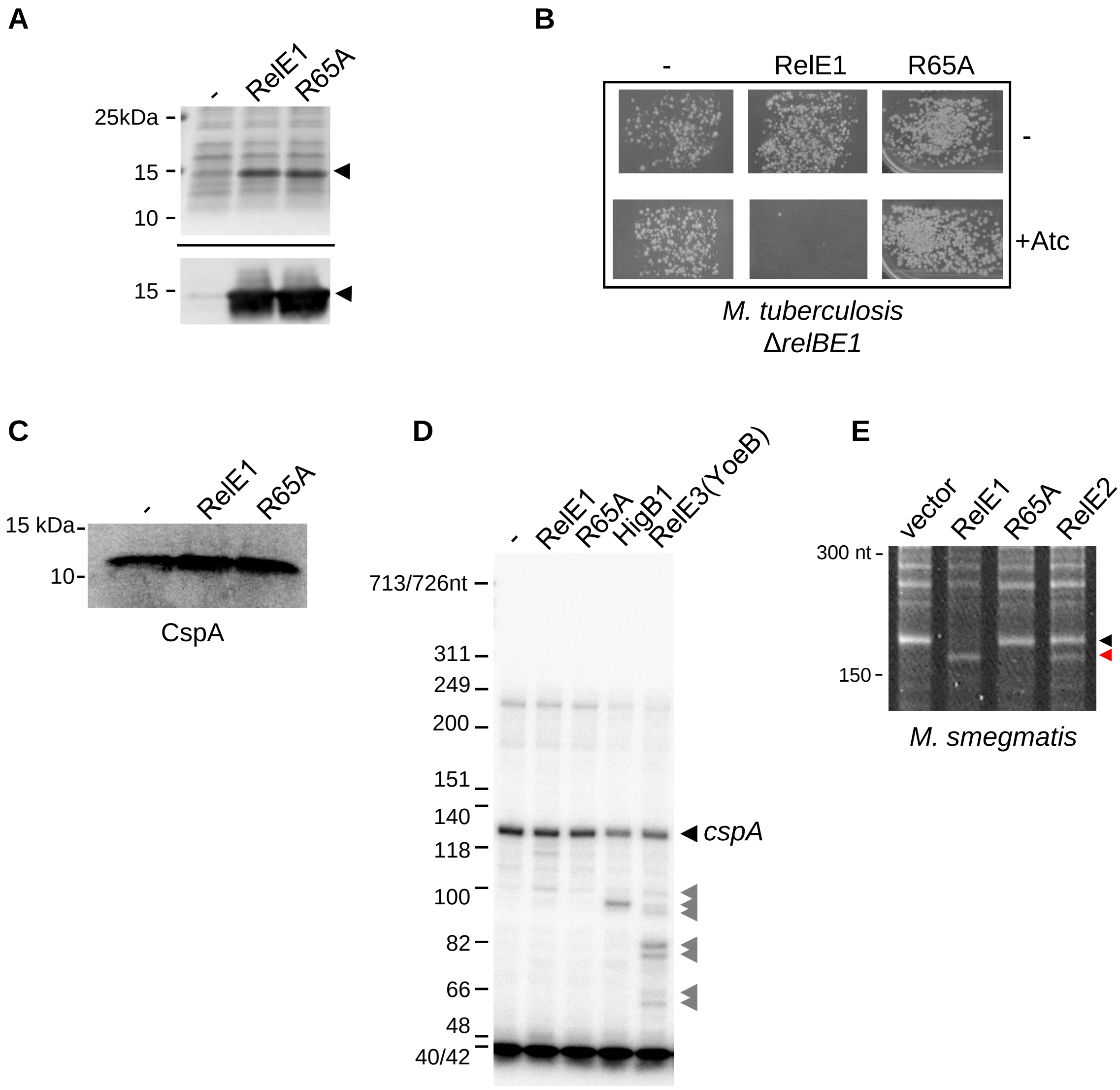

Supplementary Fig. 4: Toxicity assay in *M. tuberculosis* and *E. coli*, and *cspA* cleavage by RelE3(YoeB) *in vitro*. (**A**) Steady state levels of His-tagged RelE1 and RelE1 [R65A] expressed from in pET15b vector in E. coli BL21AI strain in the presence of 0.2% arabinose inducer overnight at 22°C. Coomassie Blue stained SDS-PAGE of whole cell extracts (top) and the corresponding western blot using anti-His antibody (bottom) are shown. (**B**) *M. tuberculosis* Δ*relBE1* mutant transformed with pGMC-vector (-), RelE1^{B1} or RelE1[R65A] were plated on 7H11 agar plates supplemented with 10% oleic acid-albumin-dextrose-catalase (OADC) with or without Atc (200 ng.ml^{-1}), and incubated for 3 weeks at 37 °C. (**C**) CspA protein synthesis is not inhibited by RelE1 or RelE1 R65A in an *E. coli in vitro* transcription/translation coupled reaction. CspA was labeled with [^{35}S]-methionine and reactions were performed for 2 h at 37 °C. Samples were separated on SDS-PAGE and visualized by phosphorimager. (**D**) RelE3(YoeB) and HigB1, but not RelE1, cleaves *cspA* mRNA *in vitro*. RNA extracted

from *M. smegmatis in vitro* translation reactions in the absence (-) or presence of toxins were subjected to primer extension with a [^{32}P]-labeled *cspA* primer. The obtained labeled cDNA was separated on denaturing urea-polyacrylamide gel and imaged by autoradiography. Arrows show the uncleaved *cspA* (126 nt, black arrow) and cleaved *cspA* (grey arrow). (**E**) RelE2 cleaves the 16S rRNA *in vivo.* RelE2, RelE1 or RelE1 R65A was expressed from a pGMC-based vector for 3 h in *M. smegmatis* in the presence of Atc inducer. Total RNA was extracted and subjected to a RNase H treatment in the presence of hybrid probe, separated on a denaturing urea-polyacrylamide gel and stained by SYBR-safe.

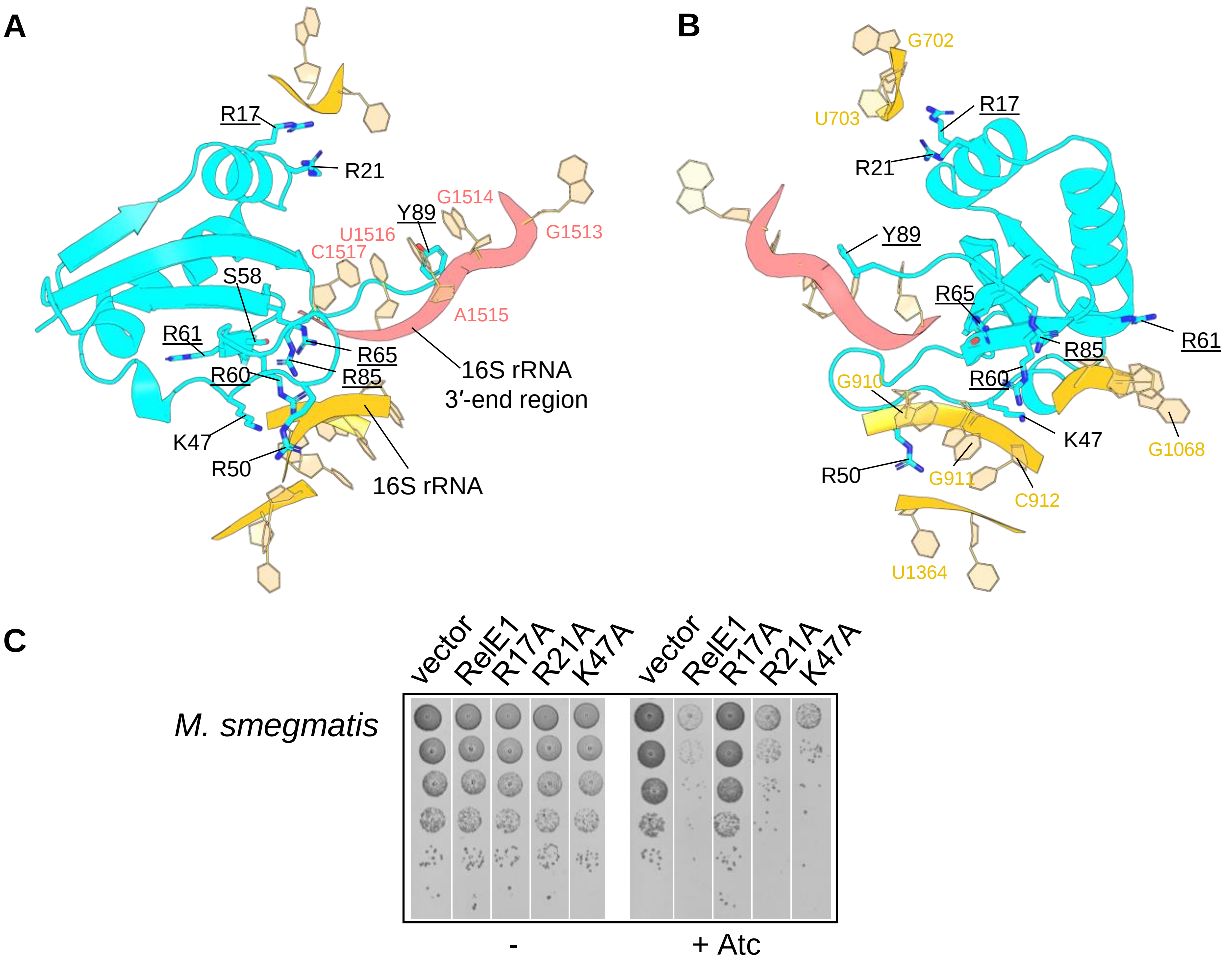

Supplementary Fig. 5: Molecular docking of RelE1 with *M. smegmatis* 70S ribosome showing RelE1 docked to the 30S subunit. (**A**) Close-up view of RelE1 as in Fig. 6D and (**B**) rotated 90 degrees about the y-axis to show residues predicted to interact with the 3′ 16S rRNA. Residues important for RelE1 activity are underlined. Ribosomal RNA and proteins of the 50S coloured teal and blue, respectively, and ribosomal RNA and proteins of the 30S coloured gold and yellow, respectively, and RelE1 coloured cyan. (**C**) Alanine substitution in putative rRNA interacting residues of RelE1. *M. smegmatis* transformed with pGMC-vector (-), RelE1^{B1} or its R17A, R21A or K47A derivatives were serially diluted, spotted on LB agar plates with or without Atc inducer at 100 ng·ml^{-1}. Plates were incubated for 3 days at 37 °C.

Contacts		RelE	RelE1	HigB1	RelE1	YoeB	RelE1
16S rRNA	h18	*K28*	-	**N73**	-	*R22*	*K25*
		K29	-	*H70*	R61	*K26*	R61
						K42	-
	h30-31	*R10*	-	*R22*	-	*K32*	-
		K13	**R17**	*K27*	**R17**	*R35*	-
		K17	R21	*K31*	R21	*R36*	-
		R93	-	*R111*	-		
	h34	*R56*	-	*N96*	-	*K21**	-
						*R22**	-
						*K25**	-
	h44	*K43*	K47	**K58**	K47	**E46**	K47
		L44	*L49*			*K49*	R50
mRNA		*K52*[b]	<u>S58</u>[b]	**K58**[b]	<u>K47</u>[b]	**E46**[b]	<u>K47</u>[b]
		**R45**	R50	E66	S58	*K49*	R50
		K54	**R60**	**R68**	**R60**	*R59*	**R60**
		**R61**	**R65**	**R77**	**R65**	**R65**	**R65**
		**R81**[a]	<u>**R85**</u>[a]	**K95**[a]	<u>**R85**</u>[a]	**H83**[a]	<u>**R85**</u>[a]
		**Y87**	**Y89**	**K99**	-	**Y84**	**Y89**

Supplementary Fig. 6: Toxin interaction with the 30S subunit. Contacts formed between the 30S subunit, target mRNAs, and *E. coli* RelE (PDB 4V7J), *M. tuberculosis* HigB1 (PDB 7NBU), or *E. coli* YoeB (PDB 4V8X). Structurally equivalent conserved residues from RelE1 are shown. Residues are shown in bold if essential for enzymatic activity, or italicized where essentiality is unknown. General acid-base pairs are underlined and superscript labels denote respective acids (a) or bases (b). Corresponding putative acid-base pairs from RelE1 are also underlined and labelled.

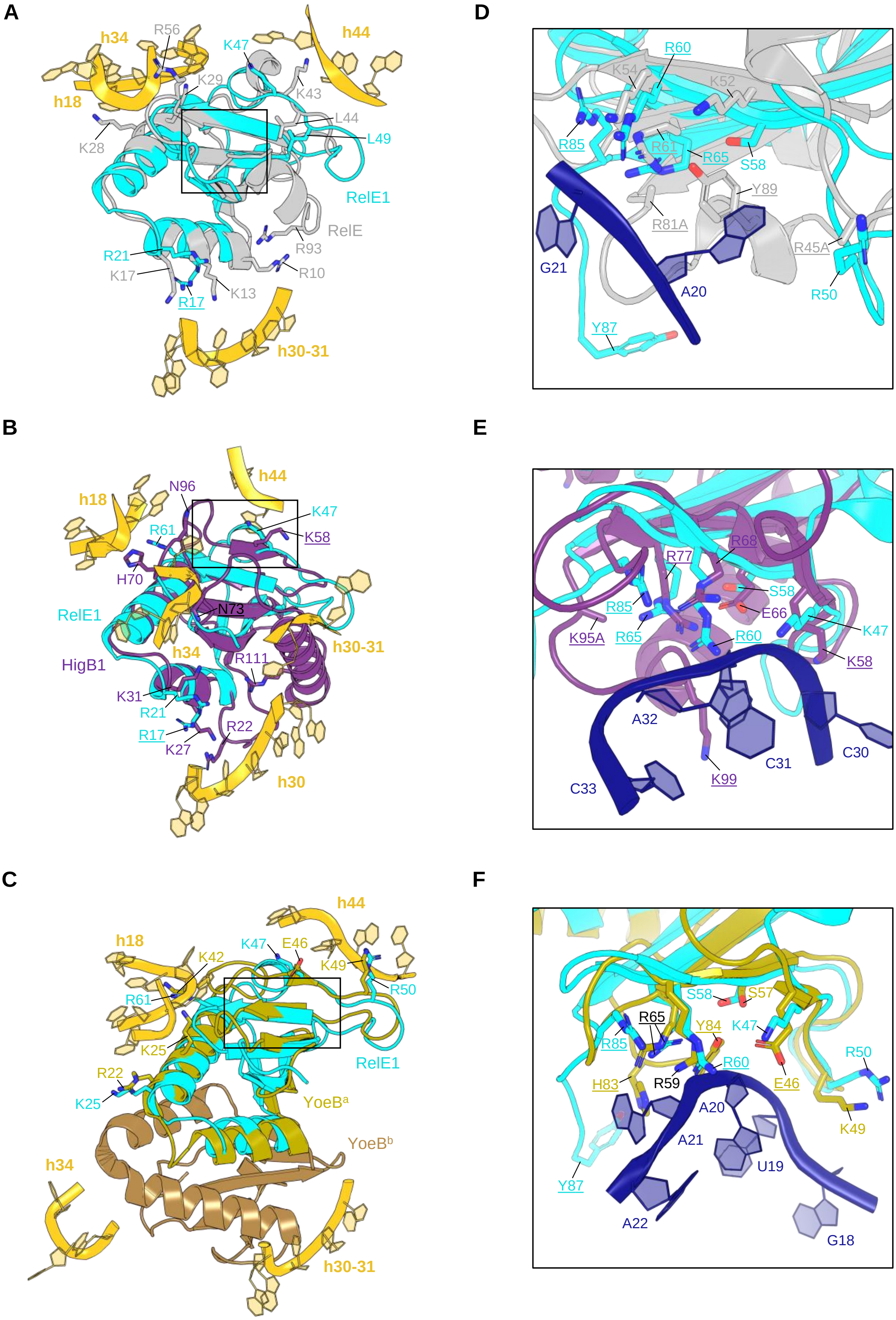
A
h34
R56
K47
h44
K29
K43
h18
L44
L49
K28
RelE1
RelE
R93
R21
R10
K17
K13
R17
h30-31
B
h44
h18
N96
K47
K58
R61
H70
RelE1
N73
h30-31
h34
R111
HigB1
K31
R21
R22
R17
K27
h30
C
h44
h18
K42
K47
E46
K49
R61
R50
K25
RelE1
R22
K25
YoeBa
YoeBb
h34
h30-31
D
R60
K54
K52
R85
R61
R65
S58
Y89
R81A
R45A
G21
A20
R50
Y87
E
R68
R77
S58
R85
E66
K47
K95A
R65
R60
K58
A32
C31
C30
K99
C33
F
S58
S57
R65
Y84
K47
R85
R50
R60
H83
R59
E46
A20
K49
A21
U19
Y87
A22
G18

Supplementary Fig. 7: Structural comparison between RelE1 and known ribosome interacting Rel toxins. Structural superpositions of *M. tuberculosis* RelE1 (PDB 9G12, cyan, this study) against (**A**) *E. coli* RelE (PDB 4V7J; RMSD = 1.80 Å across 370 atoms), (**B**) *M. tuberculosis* HigB1 (PDB 7NBU; RMSD = 1.84 Å across 374 atoms , or (**C**) *E. coli* YoeB (PDB 4V8X; RMSD = 1.53 Å across 307 atoms). 16S rRNA helices contacted by each toxin are colored gold and dark blue respectively. Interacting residues and their structural equivalents in RelE1 are shown as sticks with atoms colored red for oxygen and blue for nitrogen. Residues are underlined if essential for activity. (**D-F**) Close-up views of the boxed regions in (**A-C**) highlighting mRNA-interacting residues and their structural equivalents in RelE1. Target mRNAs are colored dark blue.

Table S1: Primers used in this work

relBE1_upstream For	CCACCTGCTACGCCGGCATGC
relBE1_upstream Rev	GATCCTCGAGACTCAGCCGAACGCCTCGTCG
relBE1_downstream For	GATCCTCGAGTAAGCCGATAAATGATGTTATGTCAAGTACGTGTTGACAATTAATCATCGGC
relBE1_downstream Rev	GATCAAGCTTACTTGACATAACATCATTTATCGGCTTATTCTCAGTCCTGCTCCTCGGC
relBE1_ZeoR For	GATCAAGCTTGCCGTACCCCGGGTACCGG
relBE1_ZeoR Rev	CGAAAACAACCCGAACACGCTG
pGMC-Infu For	CAACTTTATTATACATAGTTGATAATTC
pGMC-Infu Rev	GGGCAGCCTGTCTTCCTC
pGMC-RelE1 For	GAAGACAGGCTGCCCATGAGCGACGACCATCCCTAC
pGMC-RelE1 Rev	TGTATAATAAAGTTGTTAACGTGGCCGGCACGGGTTC
pGMC-RelB1 For	GAAGACAGGCTGCCCATGGCTGTTGTCCCAC
pGMC-RelE2 For	GAAGACAGGCTGCCCATGCCTTACACCGTGCGGTTC
pGMC-RelE2 Rev	TGTATAATAAAGTTGTTATCGGCGGTAGATGTCCG
pGMC-RelE3(YoeB) For	GAAGACAGGCTGCCCATGAGAAGCGTCAACTTCGATC
pGMC-RelE3(YoeB) Rev	TGTATAATAAAGTTGTTAGTAGTGGTATCGGGCCTTCAG
pGMC-RelE1-RelB1 For	GCCAGCCTTTCATGATCACCGCGCGGTGTAAC
pGMC-RelE1-RelB1 Rev	AAATTGGGAGATATAGAACTACCCGAGCTGCTG
pGMC-RelE1^{R17A}-B1 For	ACCGCGACAGCGGCAGCCGACCTGCAACGCT
pGMC-RelE1^{R17A}-B1 Rev	AGCGTTGCAGGTCGGCTGCCGCTGTCGCGGT
pGMC-RelE1^{R21A}-B1 For	CACGCGACCTGCAAGCCTTACCCGAAAAGATC
pGMC-RelE1^{R21A}-B1 Rev	GATCTTTTCGGGTAAGGCTTGCAGGTCGCGTG
pGMC-RelE1^{K47A}-B1 For	ACCCGCATAGGTTGGGCGCGCCGCTGCGCAATGAC
pGMC-RelE1^{K47A}-B1 Rev	GTCATTGCGCAGCGGCGCGCCCAACCTATGCGGGT
pGMC-RelE1^{R50A}-B1 For	TTGGGCAAGCCGCTGGCCAATGACCTTGAA
pGMC-RelE1^{R50A}-B1 Rev	TTCAAGGTCATTGGCCAGCGGCTTGCCCAA
pGMC-RelE1^{S58A}-B1 For	CTTGAAGGCCTCCACGCAGCCCGCCGCGGT
pGMC-RelE1^{S58A}-B1 Rev	ACCGCGGCGGGCTGCGTGGAGGCCTTCAAG
pGMC-RelE1^{R60A}-B1 For	GGCCTCCACTCAGCCGCCCGCGGTGATTAC
pGMC-RelE1^{R60A}-B1 Rev	GTAATCACCGCGGGCGGCTGAGTGGAGGCC
pGMC-RelE1^{R61A}-B1 For	CTCCACTCAGCCCGCGCCGGTGATTACCGC
pGMC-RelE1^{R61A}-B1 Rev	GCGGTAATCACCGGCGCGGGCTGAGTGGAG
pGMC-RelE1^{R65A}-B1 For	GCCGCGGTGATTACGCCGTCGTCTACGCCATCG
pGMC-RelE1^{R65A}-B1 Rev	CGATGGCGTAGACGACGGCGTAATCACCGCGGC
pGMC-RelE1^{R85A}-B1 For	ATCCACATCGCTCGTGCCAGTGCCAGCTAC
pGMC-RelE1^{R85A}-B1 Rev	GTAGCTGGCACTGGCACGAGCGATGTGGAT
pGMC-RelE1^{Y89A}-B1 For	CGTCGCAGTGCCAGCGCCCGAATGAACCCG
pGMC-RelE1^{Y89A}-B1 Rev	CGGGTTCATTCGGGCGCTGGCACTGCGACG
pGMC-V RelE infu For	AAACGCATTCTCTGACAACTTTATTATACA
pGMC-V RelE infu Rev	CAGAAAATACGCCATGGGCAGCCTGTCTTC
Rv1246c EcoRI-For	GAGAATTCCATATGAGCGACGACCATCCCTACCA (EcoRI)
Rv1246c HindIII-Rev	GAAAGCTTTTAACGTGGCCGGCACGGGT (HindIII)
Rv2866 EcoRI-For	GAGAATTCCATATGCCTTACACCGTGCGGTTC (EcoRI)
Rv2866 HindIII-Rev	GAAAGCTTGGATCCCTATCGGCGGTAGATGTCCG (HindIII)
Rv3358 EcoRI-For	GAGAATTCCATATGAGAAGCGTCAACTTCGATC (EcoRI)
Rv3358 HindIII-Rev	GAAAGCTTTCAGTAGTGGTATCGGGCCT (HindIII)

RelE1 NdeI For	TTCATATGGTGAGCGACGACCATCCCTAC (NdeI)
RelE1 BamHI Rev	TTGGATCCTTAACGTGGCCGGCACGGGTTC (BamHI)
pETduet-RelB1His For	GAGGATCCGGCTGTTGTCCCACTGGGCGAA (BamHI)
pETduet-RelB1His Rev	GAAAGCTTTCACCGCGCGGTGTAACGGTT (HindIII)
pETduet-RelB1His + RelE1 For	GACATATGAGCGACGACCATCCCTACCAC (NdeI)
pETduet-RelB1His +RelE1 Rev	GACTCGAGTTAACGTGGCCGGCACGGGTT (AvaI/XhoI)
PURE cspA For	GCGAATTAATACGACTCACTATAGGGCTTAAGTATAAGGAGGAAAAAATATGCCACAGGGAACTGTGAAG
PURE cspA Rev	AAACCCCTCCGTTTAGAGAGGGGTTATGCTAGTCAGAGGGAGCGGACTCCGGTGGCCTG
PURE gfp For	GCGAATTAATACGACTCACTATAGGGCTTAAGTATAAGGAGGAAAAAATATGGAGTAAAGGAGAAGAACTTTTCACTG
PURE gfp Rev	AAACCCCTCCGTTTAGAGAGGGGTTATGCTAGTTATTTGTAGAGCTCATCCATGCCATGTG
PURE gly For	GCGAATTAATACGACTCACTATAGGGCTTAAGTATAAGGAGGAAAAAATATGAGTGACCACGAAGTGAAG
PURE gly Rev	AAACCCCTCCGTTTAGAGAGGGGTTATGCTAGTCAGCGCTTGAGCGGGCTGTAG
cspA extension primer-1	TCTCCGTGTAGTGGACAAATACATCCGCGGAAC
cspA extension primer-2	AGCGAGCGGACTCCGGTGGCCT
gfp extension primer-1	AGGGTAAGTTTTCCGTATGTTGCATCACCTTCAC
gfp extension primer-2	TCTGCTAGTTGAACGGATCCATCTTCAATGTTGTG
gly extension primer	ACCTTGCCCGGGATGGGGTGATCGACCGGCGTG
cspA_mRNA_3’end_probe	AGCGAGCGGACTCCGGTGGCCT
gfp_mRNA _3’end_probe	ATTTGTAGAGCTCATCCATGCCA
*M.smeg*_5SrRNA_3’end _probe	AGTATCATCGGCGCTGGCAG
*M.smeg*_16SrRNA_3’end_probe	AGAAAGGAGGTGATCCAGCC
*M.smeg*_16SrRNA_Middle_probe	CTGTTCGCTCCCCACGCTTT
*M.smeg*_23SrRNA_3’end_probe	GTAAGTTTTCGGCCGGTTAG
DRNA_i7	CTGGAGTTCAGACGTGTGCTCTTCCGATCTNNNNNNNNNA
D6A	CTCTTTCCCTACACGACGCTCTTCCGATCTNTACACGGCACCAACCGAGG
D6B	CTCTTTCCCTACACGACGCTCTTCCGATCTNGTATCGGCACCAACCGAGG
D6C	CTCTTTCCCTACACGACGCTCTTCCGATCTNCGTCCGGCACCAACCGAGG
D6D	CTCTTTCCCTACACGACGCTCTTCCGATCTNAAGTCGGCACCAACCGAGG
D6E	CTCTTTCCCTACACGACGCTCTTCCGATCTNACACCGGCACCAACCGAGG
D6F	CTCTTTCCCTACACGACGCTCTTCCGATCTNGGTACGGCACCAACCGAGG
D6H	CTCTTTCCCTACACGACGCTCTTCCGATCTNTCGGCGGCACCAACCGAGG
D6I	CTCTTTCCCTACACGACGCTCTTCCGATCTNCAAGCGGCACCAACCGAGG
D6J	CTCTTTCCCTACACGACGCTCTTCCGATCTNTTGACGGCACCAACCGAGG
D6K	CTCTTTCCCTACACGACGCTCTTCCGATCTNGCTGCGGCACCAACCGAGG
D6L	CTCTTTCCCTACACGACGCTCTTCCGATCTNCCGACGGCACCAACCGAGG
D6M	CTCTTTCCCTACACGACGCTCTTCCGATCTNCTCGCGGCACCAACCGAGG
D6N	CTCTTTCCCTACACGACGCTCTTCCGATCTNAGGACGGCACCAACCGAGG
D6O	CTCTTTCCCTACACGACGCTCTTCCGATCTNATTGCGGCACCAACCGAGG

BI7RPI6_ATTGGC	CAAGCAGAAGACGGCATACGAGATATTGGCGTGACTGGAGTTCAGACGTGTGC
A-PE-PCR10	AATGATACGGCGACCACCGAGATCTACACTCTTTCCCTACACGACG
16S_RNaseH_ probe_1	ACGUAUUCACCGC<u>AGCGTT</u>GCUGAUCUGCGAUUAC
3P_V4 primer	GTATCTNNNNNNNNNNNNNNNNTGAGCCTCGGTTGGTGCCG
*M.smeg*_16SrRNA_RNA seq_3	CTGGAGTTCAGACGTGTGCTCTTCCGATCT TGAATACGTTCCCGGGCCTT
B_i7RPI2_ACATCG	CAAGCAGAAGACGGCATACGAGATACATCGGTGACTGGAGTTCAGACGTGTGC
B_i7RPI3_ACATCG	CAAGCAGAAGACGGCATACGAGATGCCTAAGTGACTGGAGTTCAGACGTGTGC
B_i7RPI7_GATCTG	CAAGCAGAAGACGGCATACGAGATGATCTGGTGACTGGAGTTCAGACGTGTGC
